# A Machine Learning-Guided Approach for Identifying Potential HCAR1 Antagonists in Lactate-Driven Cancers

**DOI:** 10.1021/acsomega.5c09253

**Published:** 2026-02-03

**Authors:** Letícia Vivas Carvalho, Núbia Seyffert, Roberto Meyer, Sandeep Tiwari, Thiago Luiz de Paula Castro

**Affiliations:** † Institute of Health Sciences (ICS), 28111 Federal University of Bahia (UFBA), Av. Reitor Miguel Calmon, S/N Canela, Salvador, Bahia 40231-300, Brazil; ‡ Institute of Biological Sciences (ICB), Federal University of Minas Gerais (UFMG), Av. Presidente Antônio Carlos 6627 Pampulha, Belo Horizonte, Minas Gerais 31270-901, Brazil

## Abstract

GPR81 (HCAR1) is a lactate-sensing G protein-coupled receptor (GPCR) involved in tumor progression, immune evasion, and therapeutic resistance across various cancers. Despite their clinical relevance and druggable nature, selective HCAR1 antagonists have yet to be identified. This study aimed to construct a statistically significant Support Vector Machine (SVM) model for binary classification (agonists versus antagonists) of HCAR1’s potential ligands and the prioritization of molecular substructures driving antagonism and receptor selectivity. An SVM model was trained on 144 ligands (66 agonists, 78 antagonists), listed in the IUPHAR/BPS Guide to Pharmacology, from 12 structurally related Class A GPCRs (HCAR1, HCAR2, HCAR3, OXER1, GPR35, SUCNR1, P2Y2, MCHR1, OPRD1, AGTR1, ADORA2A, and ADRA1A). Their ligands were encoded using physicochemical descriptors, 2048-bit ECFP4 fingerprints, and ΔAffinity scores from molecular docking to active and inactive receptor conformations. The data set was split 80/20 for training and testing, respectively, with hyperparameters (*C*, γ) being optimized via 5-fold cross-validation. SHAP analysis was performed for feature interpretation. The final SVM model achieved a test set accuracy of 79.3%, with a sensitivity of 69.2% and specificity of 87.5%. The ROC analysis yielded an AUC of 0.94, while bootstrapping confirmed robust performance with a mean AUC of 0.874 and a 95% confidence interval [0.711, 1.000]. SHAP analysis highlighted polar, rigid, and aromatic substructures as selectivity-driving features. We applied the model to screen 3,377 compounds from natural products, synthetic libraries, and FDA-approved drugs, prioritizing potential HCAR1 ligands with antagonist-like features. Based on ΔAffinity, off-target scores, and prediction confidence, Ketanserin, Cryptopyranmoscatone A1 diacetate, and Cefuroxime emerged as reference ligands with promising antagonistic potential, two of which are FDA-approved drugs. Rather than representing final hits, these molecules illustrate how structural and electronic features can favor the stabilization of inactive states in HCAR1. Overall, this work presents a proof-of-concept framework that integrates conformational docking, machine learning, and substructure interpretation to elucidate the chemical and structural determinants of HCAR1 antagonism. The findings provide fragment-level insights that may guide future bioisosteric and fragment-based design of selective antagonists for lactate-driven tumors.

## Graphical abstract

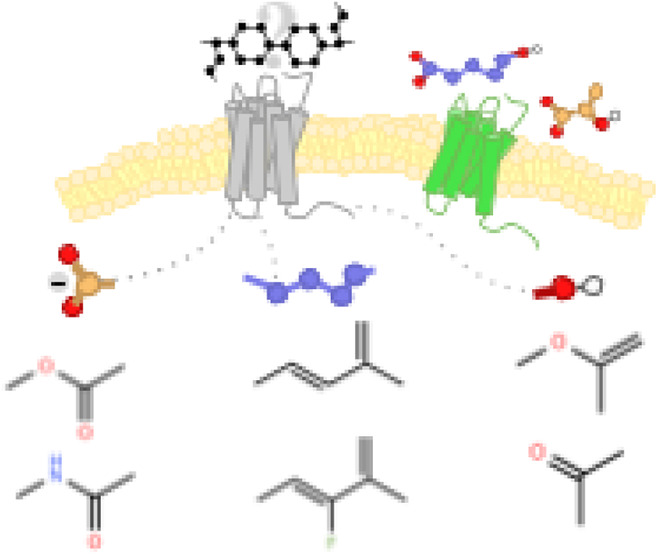

## 1 Introduction

In 2014, Roland and coworkers were the first to report the overexpression of GPR81 across multiple cancers, and both *in vitro* and *in vivo*, studied the protumorigenic role of GPR81 in aggressive malignancies, such as Pancreatic Ductal Adenocarcinoma (PDAC).^1^ Their findings paved the way for the recognition of GPR81 as an emerging cornerstone in tumor progression, particularly by supporting the tumor microenvironment through the induction of angiogenesis,^2^ upregulation of PD-L1,^3^ and increased invasiveness via ECM remodeling.^4^

Despite the growing evidence associating GPR81 expression with resistance to targeted therapies,^5–7^ only one compound—reserpine—has been experimentally proposed as a functional antagonist of GPR81/HCAR1 to date.^8^ Other compounds sometimes described as HCAR1 antagonists, such as 3-oxo-butyrate (3-OBA), do not actually block HCAR1.^9^ In addition, the structure of the only HCAR1-focused preclinical candidate announced in 2024 has not been disclosed.^10^

More than a third of FDA-approved drugs act on GPCRs, being considered the most druggable proteins. It primarily accounts for their involvement in a wide range of biological processes, their widespread localization, and functional versatility.^11^ Interestingly, out of the approximately 100 GPCRs implicated in cancer, only around 15 distinct GPCRs are currently targeted by cancer therapeutic agents, and fewer than 10 have received FDA approval for oncology indications.^12^

For decades, cancer drug discovery has focused on directly modulating tumor growth, such as inhibiting tyrosine kinases (e.g., EGFR, BCR-ABL)^13^ and growth factor receptors (e.g., HER2, VEGF),^14,15^ which are often mutated and amplified in cancer.^16^ Differently, GPCRs rarely act as classical oncogenes, thus not driving tumorigenesis. That, coupled with the initial lack of structural and functional data, turned GPCRs into less attractive targets for rational drug design.

In 2011, the hallmarks of cancer were updated to include interconnected phenomena occurring within the tumor microenvironment, including immune evasion and metabolic reprogramming.^17^ To sustain uncontrolled growth, cancer cells require a continuous supply of nucleotides, amino acids, fatty acids, and, in particular, the central energy and primary carbon source metabolite: glucose. Aerobic glycolysis is a long-term event by which even oxygen-supplied tumor cells prefer to incompletely utilize glucose over oxidative phosphorylation, leading to lactate accumulation.^18^

Later considered a metabolic waste product, lactate is now well established as the endogenous agonist of GPR81, also known after deorphanization as the Hydroxycarboxylic Acid Receptor 1 (HCAR1).^19^ Upon lactate binding, HCAR1 undergoes a multilayered allosteric switch that leads to protein Gi dissociation into Gαi and Gβγ, which, by binding to effector proteins, prompt downstream signaling pathways that ultimately regulate gene expression. The extensive structural and functional exploitation of HCAR2, its closest sibling (identity >52%), for the development of more selective antidyslipidemic drugs has opened the avenue to a closer understanding of the HCAR1 activation mechanism, as highlighted by the recently reported Cryo-EM of HCAR1 coupled with the selective agonist 3Cl-5OH-BA.^20^

Given the central yet underexplored role of GPR81/HCAR1 as a therapeutic target, we propose a rational, early-stage drug discovery strategy to identify potential HCAR1 antagonists.

Recent AI tools, such as AiGPro, DeepGPCR, EnGCI, and GPCRVS, have improved large-scale GPCR ligand prediction.^21–24^ However, these deep learning models rely on large, curated data sets, which are less suitable with the limited number of ligands available for HCAR1.^25^ Notably, the few studies that classified agonists and antagonists at the single-receptor level successfully used Support Vector Machines (SVMs) for the 5-HT_1A_ serotonin receptor^26^ and the thyroid hormone receptor.^27^

Given the above-mentioned, by combining ensemble molecular docking with machine learning techniques and building on successful previous works,^26,27^ we developed a Support Vector Machine (SVM) classifier capable of accurately distinguishing between agonists and antagonists of HCA-related GPCRs based on docking-derived binding affinities, physicochemical descriptors, and ECFP4 molecular fingerprints.

A virtual screening protocol guided by the trained machine learning model was applied to a library of 3,733 compounds from diverse origins (natural products, synthetic molecules, and FDA-approved drugs). Docking simulations against both the active and inactive conformations of HCAR1 enabled the prioritization of three compounds with high predicted antagonistic potential and low likelihood of cross-reactivity with other Class A GPCRs included in the data set.

Furthermore, rather than being treated as final hits, the prioritized molecules were used as chemical references to understand what defines antagonism at the HCAR1 level. By examining their docking profiles and molecular fingerprints, in light of the SVM model findings, we identified consistent patterns of features potentially linked to antagonist prediction and receptor selectivity. Altogether, these findings provide a foundation for fragment-based design, following the same rationale successfully applied in the development of other GPCR-targeted anticancer agents, such as the CXCR4 antagonist HF51116 ^28^ and A_2A_ receptor antagonists.^29^

## 2 Materials and Methods

### 2.1 Data Set Construction

A total of 144 ligands (66 agonists and 78 antagonists of 12 Class A GPCRs) curated from the IUPHAR/Guide to Pharmacology comprised the data set (Table 1). Given our intent to apply the binary classifier to HCAR1-predicted ligands, we initially prioritized receptors structurally and/or functionally similar to the target, such as HCAR2, HCAR3, OXER1, and GPR35.^30^ However, the scarcity of known antagonists among those led to the inclusion of additional Class A GPCRs to ensure data balance (Figure 1).

**Table 1:** Number of Agonists and Antagonists for Each G Protein-Coupled Receptor (GPCR) Included in the Data set^a^

Name	No. of agonists	No. of antagonists	Total No.
HCAR1	3	0	3
HCAR3	9	0	9
HCAR2	16	0	16
OXER1	2	1	3
GPR35	6	0	6
P2RY2	0	1	1
AGTR1	0	13	13
SUCNR1	1	2	3
OPRD1	16	7	23
MCHR1	0	6	6
ADRA1A	0	32	32
ADORA2A	13	16	29
Total	66	78	144

a Receptors include Hydroxycarboxylic Acid Receptors (HCAR1–3), Oxoeicosanoid Receptor 1 (OXER1), GPR35, P2Y Purine Receptor 2 (P2RY2), Angiotensin II Receptor Type 1 (AGTR1), Succinate Receptor 1 (SUCNR1), Delta Opioid Receptor (OPRD1), Melanin-Concentrating Hormone Receptor 1 (MCHR1), and Adenosine Receptors (ADRA1A and A2AR).

**Figure 1:**
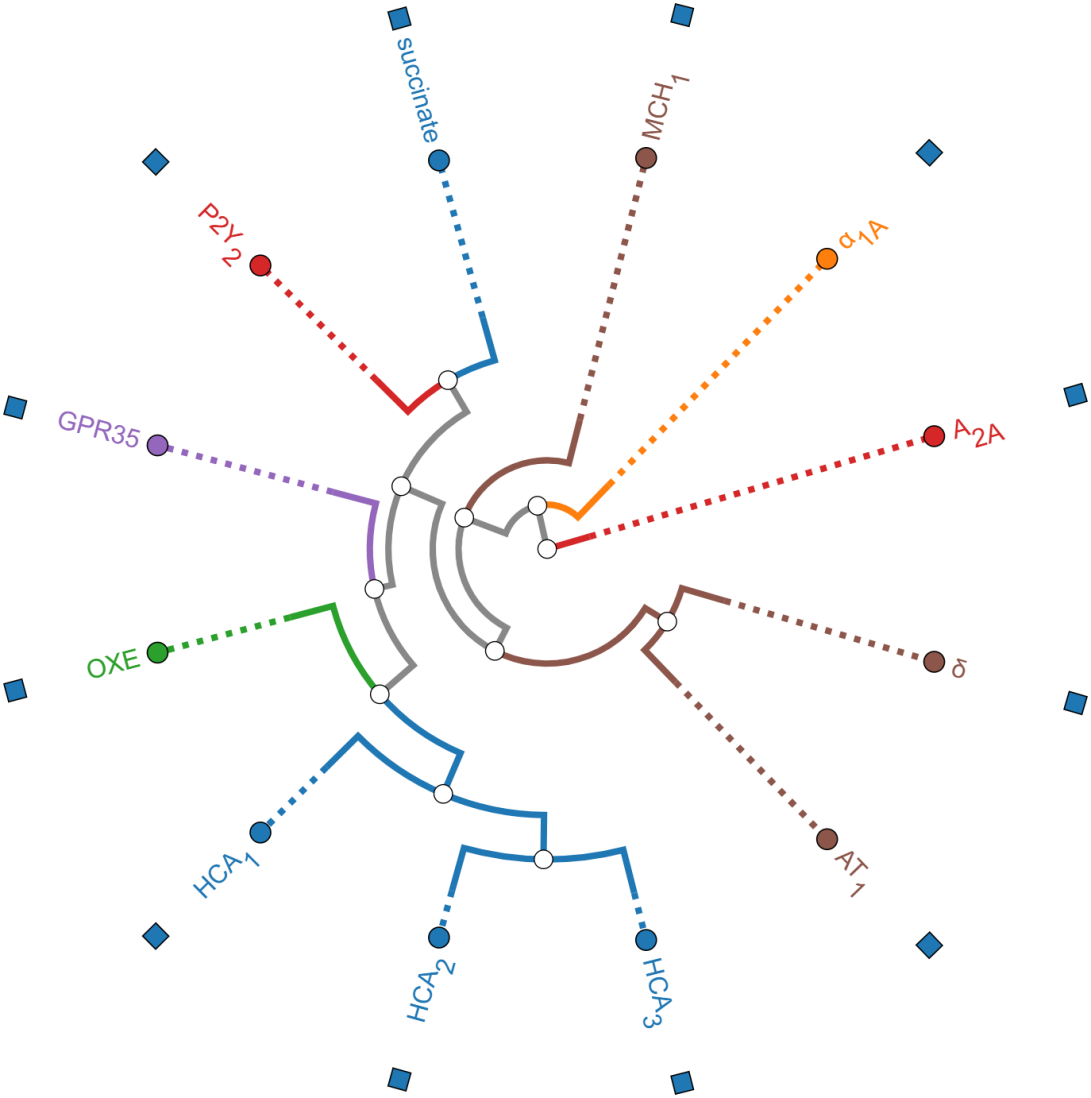
Phylogenetic relationships among Class A GPCRs used for model training and off-target assessment. Receptor branches are color-coded by subfamily or functional class. Blue: metabolic receptors, such as hydroxycarboxylic acid receptors (HCAR1, HCAR2, HCAR3) and succinate receptor (SUCNR1). Green: Oxoeicosanoid receptor (OXE). Purple: Orphan GPCR (GPR35). Red: Purinergic (P2Y2) and adenosine (A_2A_) receptors. Orange: Adrenergic α_1A_ receptor. Brown: Neuropeptide and peptide hormone receptors (MCHR1, δ-opioid, angiotensin AT1). The circular dendrogram was generated using the GPCRdb platform based on receptor sequence alignment of the selected Class A GPCRs.

All primary sequences—exclusively from *Homo sapiens*—were obtained from the UniProt Database and aligned to human (taxid: 9606) PDB proteins using BLASTp.^31^ The UniProt identifiers and taxonomic information for all GPCR sequences used in this study are provided in Table S1. For HCAR1 specifically, we used the human ortholog (UniProt ID: Q9BXC0), and all templates or modeled conformers were selected based on the highest identity to the human receptor to avoid known species-dependent pharmacological differences.

The agonist-bound and/or G-protein-coupled resolved structures were classified as active conformers. In contrast, the inactive state and antagonist- or inverse agonist-bound structures were classified as inactive conformers. Highest-resolution (lower Å) conformers were retrieved from PDB (Table S2).^32^ In the absence of one or both conformers, receptors with greater than 25% sequence identity served as templates for SwissModel homology modeling (Table S2).^33^ Only models with ≥85% of residues in favored Ramachandran regions were accepted (Table S3).^34^

Receptors were prepared with UCSF Chimera (DockPrep)^35^ and AGFR’s 1.2 prepare_receptor.py,^36^ by adding hydrogens and charges, respectively. Grid boxes (25 Å^3^) were centered based on cocrystallized ligands or literature-reported orthosteric residues using Chimera’s AutoDock Vina tool (Tables S4 and S5). Additionally, for HCAR1 active and inactive conformers, the binding site properties were evaluated using DogSiteScorer.^37^ Pocket volume, surface area, and depth measurements were stored for interpretation purposes.

Preferentially, the tridimensional SDF conformers of the ligands with no more than one violation of Lipinski's rule were retrieved from PubChem,^38^ then processed with OpenBabel 2.4.0:^39^ 3D generation (if needed), UFF minimization (1500 steps), pH 7.4 hydrogenation, and PDBQT conversion.

Molecular docking simulations were conducted in batch mode with AutoDock Vina 1.2.6 ^40^ on both receptor conformers. To guarantee optimum docking performance, the exhaustiveness parameter was set to 25.^41^ Differential binding affinities of best poses were calculated, and ligands with ΔAffinity (eq 1) within a ±0.3 kcal/mol interval were excluded, except for the endogenous ligand L-lactate, which was retained given its physiological relevance in HCAR1 activation.

ΔAffinity is the difference between the predicted binding free energy of the active conformation and the inactive conformation. Positive ΔAffinity values indicate preferential binding to the inactive state, consistent with potential antagonist activity.

1 $$\Delta {\mathrm{Affinity}}_{r}=\Delta {G\mathrm{active}}_{r}-\Delta {G\mathrm{inactive}}_{r}$$

### 2.2 Feature Engineering

The chemical and physical properties of the 144 ligands were retrieved from the PubChem database. The following descriptors were selected as features for machine learning: Molecular Weight (g/mol), Log*P* (computed by XLogP3-AA), Hydrogen Bond Donor Count (HBD), Hydrogen Bond Acceptor Count (HBA), Topological Polar Surface Area (TPSA; Å^2^), and the ΔAffinity values derived from molecular docking scores using the *Vina* scoring function.

In addition, the SMILES representations of all ligands were retrieved from PubChem and used to generate Extended-Connectivity Fingerprints (ECFP4) using RDKit v2025.03.2 ^42^ (installed via conda-forge) with Morgan fingerprinting. To balance specificity and generalizability, a radius of 2 was employed, capturing atomic environments up to two bonds away from the central atom. It resulted in a 2048-bit binary vector for each molecule. These binary fingerprints encode the presence or absence of circular substructures centered around each atom.

The final feature set thus combined physicochemical descriptors, ΔAffinity values, and molecular fingerprints. Ligands annotated as agonists were assigned the label “0”, and antagonists were labeled as “1” for classification tasks.

### 2.3 SVM Model Development

The dataset was randomly divided stratified into training (80%) and test (20%) subsets using random_state = 42 to ensure reproducibility (Table 2). Since Support Vector Machines (SVMs) rely on distance-based computations, feature scaling is essential to prevent features with larger ranges, such as Molecular Weight (MW), from dominating those with smaller ranges, like Log*P*. To address this, all features were standardized using StandardScaler, which normalized the data to a mean of 0 and a standard deviation of 1. The scaler was fitted on the training data and subsequently applied to scale the test set using the same parameters to avoid data leakage.

**Table 2:** Stratified Distribution of Agonists and Antagonists across Training and Test Sets

Data set	No. of agonists	No. of antagonists	Total No.
Training set	53	62	115
Test set	11	18	29

Hyperparameter tuning and model training were performed using scikit-learn v1.6.1 ^43^ in a Python 3.11.12 environment. The former was conducted using a stratified 5-fold cross-validation approach. A grid search was applied to explore combinations of the regularization parameter *C* (values: 0.1, 1, 10), the kernel coefficient gamma (“scale,” “auto”), and the Radial Basis Function (RBF) kernel. During each fold, four subsets were used for training and one for validation, ensuring that class distribution was preserved across all splits.

The model was then retrained on the full training set using the best hyperparameter combination identified by the grid search. It was subsequently evaluated on the independent test set by generating both class label predictions and the corresponding probabilities of antagonism. Model performance was assessed using a classification report, which provided precision, recall, F1-score, and support for each class, as well as a confusion matrix that offered insight into the model’s ability to distinguish agonists from antagonists.

### 2.4 Model Evaluation

To assess model performance and its statistical significance, we conducted 5-fold cross-validation using the original class labels and computed the mean accuracy. To determine whether this result could occur by chance, a permutation test was performed by randomly shuffling the class labels 1,000 times and recalculating cross-validated accuracy for each permutation. An empirical *p*-value was estimated as the fraction of permutations yielding an accuracy equal to or greater than the original. Statistical significance was set at *p* < 0.05 (i.e., <50/1,000 permutations).

Model discrimination was further evaluated using the Area Under the ROC Curve (AUC) on the test set, based on predicted probabilities. To estimate the robustness and variability of the AUC, we applied bootstrap resampling (1,000 iterations), where each iteration used a test set sample drawn with replacement. A 95% confidence interval was derived, and the AUC distribution was visualized via a histogram.

To interpret model predictions, we applied SHAP (v0.47.2) for feature attribution.^44^ The input data were rescaled with StandardScaler, and the first 50 ligands were selected for SHAP analysis, retaining their unscaled values for descriptor readability. Since SVM lacks a native SHAP explainer, KernelSHAP was used with a background set of 10 representative ligands selected via *K*-means clustering.

SHAP values were computed for all samples and separated into physicochemical descriptors and fingerprint features. Summary plots visualized feature importance, and the top five fingerprint bits with the highest mean absolute SHAP values were identified. Corresponding substructures activating each bit were retrieved and visualized using RDKit’s Draw.MolsToGridImage.

### 2.5 Substructure-Based Filtration

The compound library to be docked against HCAR1 was curated from three databases with different scopes: (i) NuBBEdb, a source of natural products from Brazilian biodiversity;^45^ (ii) BraCoLi, harboring synthetic compounds developed by Brazilian research groups;^47^ (iii) the FDA-approved drugs catalog of Enamine Database (library code FAD-1123, November 2023).

The entire BraCoLi drug collection (1,176 compounds) and the Enamine catalog (1,123 compounds) were downloaded and converted to SMILES format using OpenBabel v2.4.0. The same was done to the NuBBEdb compounds that did not violate Lipinski’s rule (1,434 compounds). At that time, the compound library totaled 3,377 compounds.

In the Jupyter Notebook RDKit environment, the top 5 molecular fingerprints predicted as antagonism drivers by the SVM model were used to filter the 3,377 compounds, which were then further converted from SMIs into SDF files. The preparations of the filtered compounds, which included energy minimization using the Universal Force Field, hydrogen addition based on physiological pH (7.4), and conversion to pdbqt format, were conducted with Open Babel v2.4.0 via its command-line interface.

### 2.6 Dual-Conformation Docking against Active and Inactive HCAR1 States

By using AutoDock Vina 2.7.0, the prepared compound library was docked in batch mode with HCAR1 active and inactive conformers. The active conformer was extracted from the 3.16 Å resolved Cryo-EM structure of the human HCAR1-Gi complex with 3-chloro-5-hydroxybenzoic acid (PDB 9IZD).^20^ The inactive conformer was modeled using SwissModel^33^ with the 2.7 Å resolved crystal structure of HCAR2 (PDB 7ZLY) as the template.

Receptor preparation followed the same protocol described in Section 2.1, as well as the grid box size. Its center coordinates were set according to the CHBA orientation for the active conformer and the coordinates of the corresponding orthosteric binding residues for the inactive conformation.

The positive differential binding affinities (only best poses) of nonborderline (>0.3 kcal/mol) compounds were inserted in an Excel file along with SMILES strings, and the exact physical-chemical properties were accounted for in the SVM model. These were predicted by SwissADME,^46^ using XLogP3 as the Log*P* computer, which is consistent with the SVM data set (Section 2.1).

### 2.7 Scoring and Selection of High-Confidence Antagonist Candidates

The selected ligands’ table was downloaded as a comma-separated value file and imported, along with the feature scaler and trained SVM classifier, into a Jupyter Notebook to generate molecular fingerprints and reliably predict antagonism. To this end, fingerprint radius and lengths were set in accordance with the SVM model (Radius = 2; NBits = 2048), and only the 10% most confidently predicted antagonists were selected based on the confidence percentile ranking of the confidence scores. This approach optimizes the prioritization of the most confident predictions by ranking each ligand relative to others, thereby avoiding thresholding issues that can occur when using only absolute confidence scores.

### 2.8 Promiscuity Estimation Using Docking-Derived Off-Target Scores

The conservation of the orthosteric pocket among GPCRs and the composition of our SVM model raised concerns about promiscuous binding and biased antagonism. To evaluate this hypothesis, we performed molecular docking simulations between the 10% high-confidence ligands and the Class A GPCRs used to construct the model. The simulations were conducted in AutoDock Vina 1.2.6. Following Section 2.1.

The raw binding affinities for active and inactive conformers of the receptors (including HCAR1) were applied to a Jupyter Notebook script that initially computed ΔAffinity for each receptor. The sum of the absolute ΔAffinities for all receptors other than HCAR1 represented the Off-Target Score (eq 2). Ligands with the lowest off-target scores and higher HCAR1 ΔAffinity (preferential binding to the inactive conformer) were submitted to tridimensional and two-dimensional evaluation of interactions with HCAR1 through UCSF Chimera 1.16 ^48^ and ProteinPlus (PoseView tool),^48,49^ respectively.

The OffTarget Score is calculated as the sum of the absolute ΔAffinity values across all off-target GPCRs (r ≠ HCA1). This score reflects the ligand’s potential for nonspecific interactions, with higher values indicating greater promiscuity.

2 $$\mathrm{OffTarget Score}=\underset{r\neq HCA1}{\sum }\left|\Delta {\mathrm{Affinity}}_{r}\right|$$

### 2.9 Fingerprint Correlation with GPCR Selectivity Profiles

By using RDKit, the prioritized ligands with annotations for OffTarget Score and ΔAffinity were featurized to Morgan fingerprints (ECFP-like, radius = 2, nBits = 2048). To avoid meaningless correlations, bits present in all or none of the ligands were filtered out. The remaining (informative bits) were used to create a binary presence/absence vector across the prioritized ligands. Imported from *scipy.stats*, Spearman’s rank correlation was computed between this vector and the OffTarget Score. Considering absolute correlation “r”, the top fingerprint bits were selected and translated into approximate SMILES representations using RDKit’s substructure query tools. Finally, using Seaborn, we generated a horizontal bar plot of the top fingerprint bits ordered by correlation magnitude and colored by direction of association (positive = higher off-target risk; negative = selective).

### 2.10 Target-Specific Validation Using Known HCAR1 Agonists

To assess the model’s generalization to receptor-specific ligands, all six experimentally confirmed HCAR1 agonists listed in the IUPHAR database were evaluated using the trained SVM classifier. Although belonging to the original data set, three of these compounds—L-lactate, 3,5-dihydroxybenzoic acid (3,5-DHBA), and the reference GPR81 agonist 1—were reanalyzed to verify prediction consistency. The remaining ligands, D-lactic acid, 4-hydroxybutanoate (γ-hydroxybutyrate), and nicotinic acid, were retrieved from PubChem as 3D SDF conformers, along with their physicochemical properties (MW, Log*P*, HBA, HBD, TPSA). After ligand preparation with OpenBabel 2.4.0 as described in Section 2.1, each compound was docked to both receptor conformations to compute ΔAffinity (eq 2). Application of the SVM model yielded the predicted class (agonist or antagonist), prediction probability, and confidence score.

### 2.11 Design of HCAR1 Analogs Containing Antagonist-Driver Fragments, Docking, and Surface Accessibility Analysis

To evaluate the impact of the top antagonist-driver fragments on molecular interactions with HCAR1, we designed analogs of its known agonists that bear these fragments. L-lactate and γ-hydroxybutyrate were selected as templates due to their small size and the lowest agonist prediction probability (87.5%), respectively, as reported in the Results. The nonacidic hydroxyl group common to both molecules was chosen as the substitution site.

The analogs were manually constructed in the Marvin JS extension of SwissADME by replacing this hydroxyl group with the most influential antagonist-associated fragments—a methyl ketone (bit 370) and an isopropenoxy (bit 249), represented in Figure 3. The resulting structures were exported as SMILES strings, converted into three-dimensional conformers, and prepared for molecular docking using OpenBabel v2.4 (see Section 2.1).

Molecular docking was performed with AutoDock Vina for both the active (PDB 9IZD) and inactive (homology model from PDB 7ZLY) conformations of HCAR1, and the best poses were inspected in UCSF Chimera.

To complement the visual inspection, solvent-accessible surface areas (SASA) were computed for the best docking poses, isolated receptors, and receptor–ligand complexes using Discovery Studio Visualizer v21.1.0.20298.^50^ Calculations employed a probe radius of 1.4 Å (approximating a water molecule) and 240 grid points per atom. SASA estimates the molecular surface area accessible to solvent molecules by translating a spherical probe across the van der Waals surface to map solvent-exposed regions.^51^ Although SASA is more commonly applied in molecular dynamics (MD) simulations to monitor solvent accessibility and complex stability over time,^52,53^ here it was adapted as a static comparative descriptor to quantify ligand accommodation across the active and inactive states of HCAR1 (eq 7). In this sense and following the same rationale as for ΔAffinity (eq 2), the difference in solvent exposure between receptor conformations was expressed as Differential SASA (eq 7). Finally, all designed analogs were submitted to the SVM model after calculation of physicochemical descriptors (MW, Log*P*, HBA, HBD, and TPSA) using SwissADME.

ΔSASA was calculated as the difference between the combined solvent-accessible surface areas (SASAs) of the unbound receptor and the ligand’s best docking pose and the SASA of the resulting complex. This value represents the portion of solvent-exposed area lost upon binding—essentially, the degree of ligand burial within the receptor.

3 $$\Delta \mathrm{SASA}=\left({\mathrm{SASA}}_{receptor}+{\mathrm{SASA}}_{ligand}\right)-{\mathrm{SASA}}_{complex}$$

Differential SASA compares the buried area between receptor conformations; positive values mean the ligand is more deeply buried in the inactive state, suggesting it may stabilize that conformation in a way consistent with antagonist behavior.

4 $$\mathrm{Differential}SASA=\Delta {\mathrm{SASA}}_{inactive}-\Delta {\mathrm{SASA}}_{active}$$

## 3 Results

### 3.1 Model Performance

Hyperparameter tuning with 5-fold cross-validation yielded an estimated accuracy of 0.756 using the best parameters (“C”: 10, “gamma”: “auto”, “kernel”: “rbf”). These were used to train the final model, which achieved high accuracy on the test set. The SVM classifier showed consistent performance across agonist and antagonist classes (F1 score = 0.79). Test evaluation yielded an AUC of 0.94 (Figure 2A), improving upon the initial cross-validated AUC of 0.88. However, the model demonstrated better recall for antagonists (specificity = 88%) than for agonists (sensitivity = 69%) based on the confusion matrix (eqs 3–6; Figure 2B), suggesting a mild bias toward antagonist classification. Given the study’s focus on antagonist screening, this is not considered a major limitation. Permutation testing (*n* = 1,000) yielded an empirical *p*-value of 0.00 and a cross-validation accuracy of 0.7652, supporting statistical significance (*p* < 0.05) (Figure 2C). Bootstrap resampling of the test set (1,000 iterations) further demonstrated model robustness, with a bootstrapped AUC of 0.874 and a 95% confidence interval [0.711–1.000] (Figure 2D).

Accuracy was 0.793, indicating that 79.3% of the test set’s ligands were correctly classified either as agonists or antagonists.

$$\mathrm{Accuracy}=\frac{TP+TN}{TP+FP+TN+FN}=\frac{9+14}{9+2+14+4}=\frac{23}{29}≅0.793$$ 5

Sensitivity was 0.692, indicating that 69.2% of the test set’s agonists were correctly classified as agonists.

6 $$\mathrm{Sensitivity}=\frac{TP}{TP+FP}=\frac{9}{9+4}=\frac{9}{13}≅0.692$$

Specificity was 0.875, indicating that 87,5% of the test set’s antagonists were correctly classified as antagonists.

$$\mathrm{Specificity}=\frac{TN}{TN+FP}=\frac{14}{14+2}=\frac{14}{16}=0.875$$ 7

**Figure 2:**
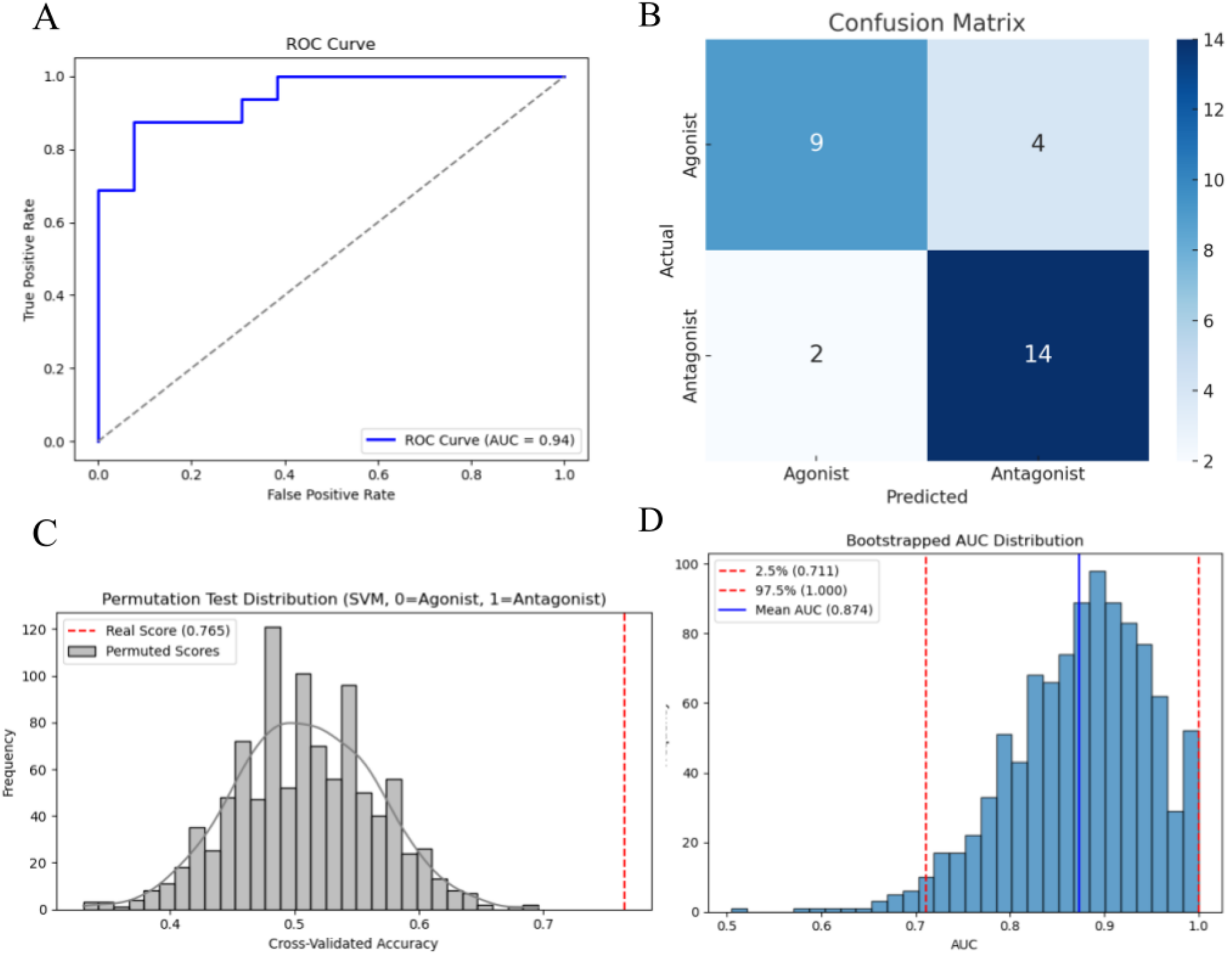
Model performance plots. (A) Receiver operating characteristic (ROC) curve showing the classifier performance on the test set (AUC = 0.94). (B) Confusion matrix of the support vector machine classifier evaluated on the test set, with true positives (TP = 9) and true negatives (TN = 14) highlighted along the diagonal. (C) Permutation test for cross-validated accuracy obtained from 1,000 permutations. The observed cross-validation accuracy (red dashed line) was 0.765, lying outside the distribution of permuted scores and yielding an empirical *p*-value of 0.00. (D) Bootstrapped distribution of the AUC based on 1,000 resamples from the test set predictions. The mean bootstrapped AUC was 0.874, with a 95% confidence interval of [0.711, 1.000].

### 3.2 Feature Interpretability

As shown in the SHAP summary plot (Figure 3), physicochemical descriptors had a minimal influence on the model predictions, except for Molecular Weight, which, at high values, modestly pushed the prediction toward antagonism. All other descriptors (Log*P*, HBD, HBA, TPSA, and ΔAffinity) showed no significant impact on model output, clustering around zero SHAP impact (Figure 2A). Although marginal, it is worth noting that more hydrophilic molecules slightly leaned toward agonist prediction. In contrast, the molecular fingerprints proved to be the main drivers of model performance, with many positive SHAP value-fingerprint bits (e.g., 896, 249, 497, 370, and 1017) highly increasing antagonist classification probability (Figure 3B). Figure 3C illustrates the molecular fragments encoded by these top-ranking bits. Notably, among them, the presence of methyl ketone (bit 370) had the greatest impact on antagonism prediction (SHAP value > 0.175).

**Figure 3:**
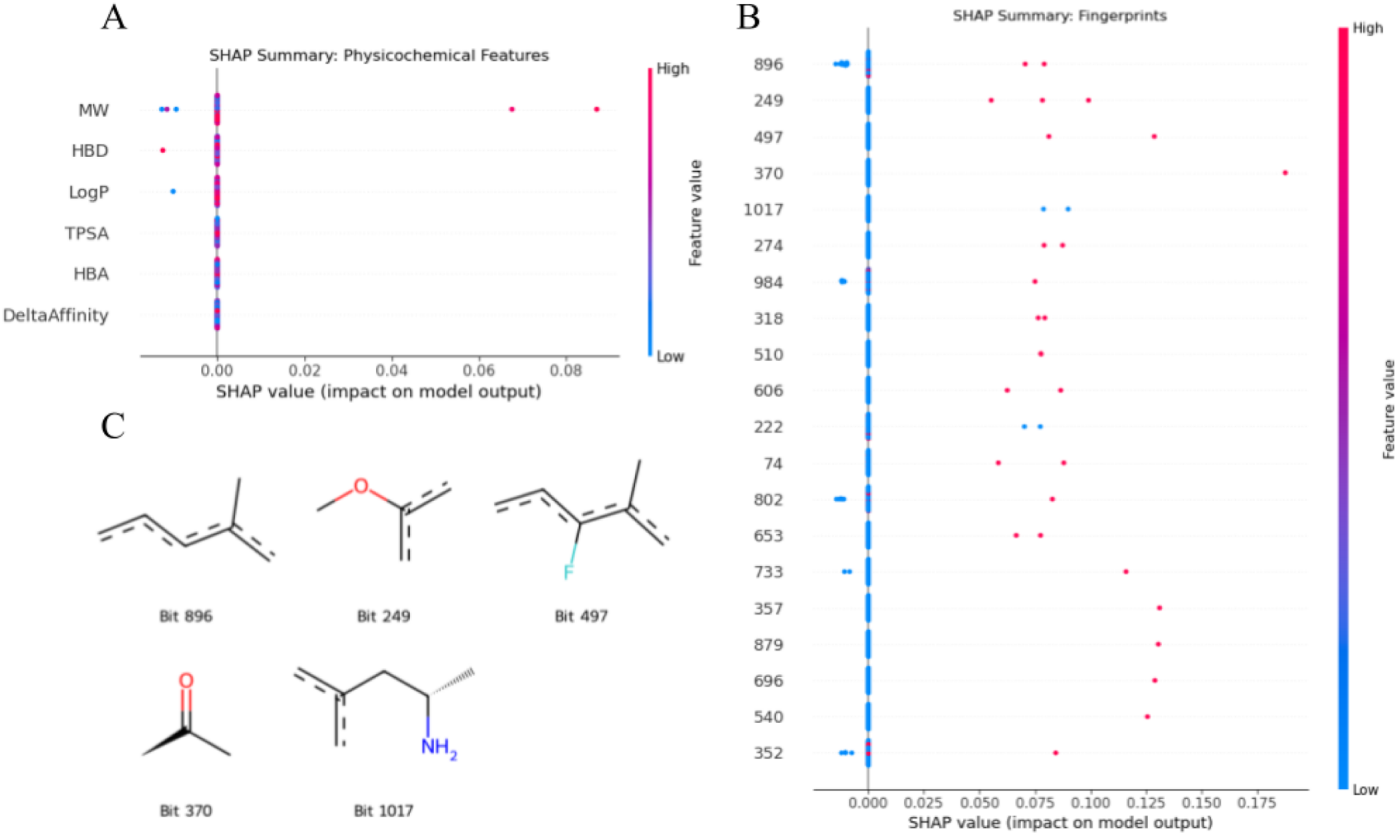
SHAP summary plots and substructure visualization of top fingerprint bits. (A) SHAP impact of physicochemical descriptors on model output. Most descriptors, including Log*P*, HBD, HBA, TPSA, and ΔAffinity, clustered around zero, indicating negligible influence on classification. Molecular weight (MW) was the only descriptor with a modest positive impact, where higher values slightly increased the probability of antagonism. (B) SHAP summary plot of molecular fingerprints. Fingerprint bits contributed significantly to model predictions, with several (e.g., bits 896, 249, 497, 370, and 1017) associated with high SHAP values and a strong influence on antagonist classification. (C) Substructure visualization of top fingerprint bits. These fragments were extracted from molecules corresponding to the most impactful bits in panel B. Notably, bit 370, corresponding to a methyl ketone moiety, had the highest influence on antagonism prediction (SHAP > 0.175), followed by isopropenyl ether (bit 249), fluorinated alkene (bit 497), and amino alkenes (bit 1017).

### 3.3 Filtering and Screening Outcomes

Application of the top 5 fingerprints associated with the antagonist behavior (e.g., 896, 249, 497, 370, and 1017) to filter the 3,377 compound library resulted in 895 filtered compounds with a notably higher proportion of hits originated from the NuBBE natural products database (731 out of 1,434 compounds; 50.10%) compared to the synthetic Enamine (143 out of 1,123; 12.73%) and BraCoLi-derived libraries (21 out of 1,176; 1.79%). This discrepancy suggests that natural products exhibit greater structural diversity, which is compatible with GPCR antagonism profiles, possibly due to their intrinsic complexity and evolutionary tuning toward protein–ligand interactions. Out of the 895 filtered compounds, 87 compounds with greater affinity toward the HCAR1 inactive conformer (>0.3 kcal/mol) were selected as potential antagonists.

### 3.4 High-Confidence Antagonist Candidates

All selected 87 compounds were classified as antagonists (label 1) upon application to the SVM model. As shown in Figure 4, the confidence scores varied across ligands, with most predictions centered around a score of ∼0.65. Out of the 87 compounds, nine scored above the 90th percentile threshold of 0.75 (indicated by the red dashed line), being considered the top 10% most confidently predicted antagonists (Table 3).

**Figure 4:**
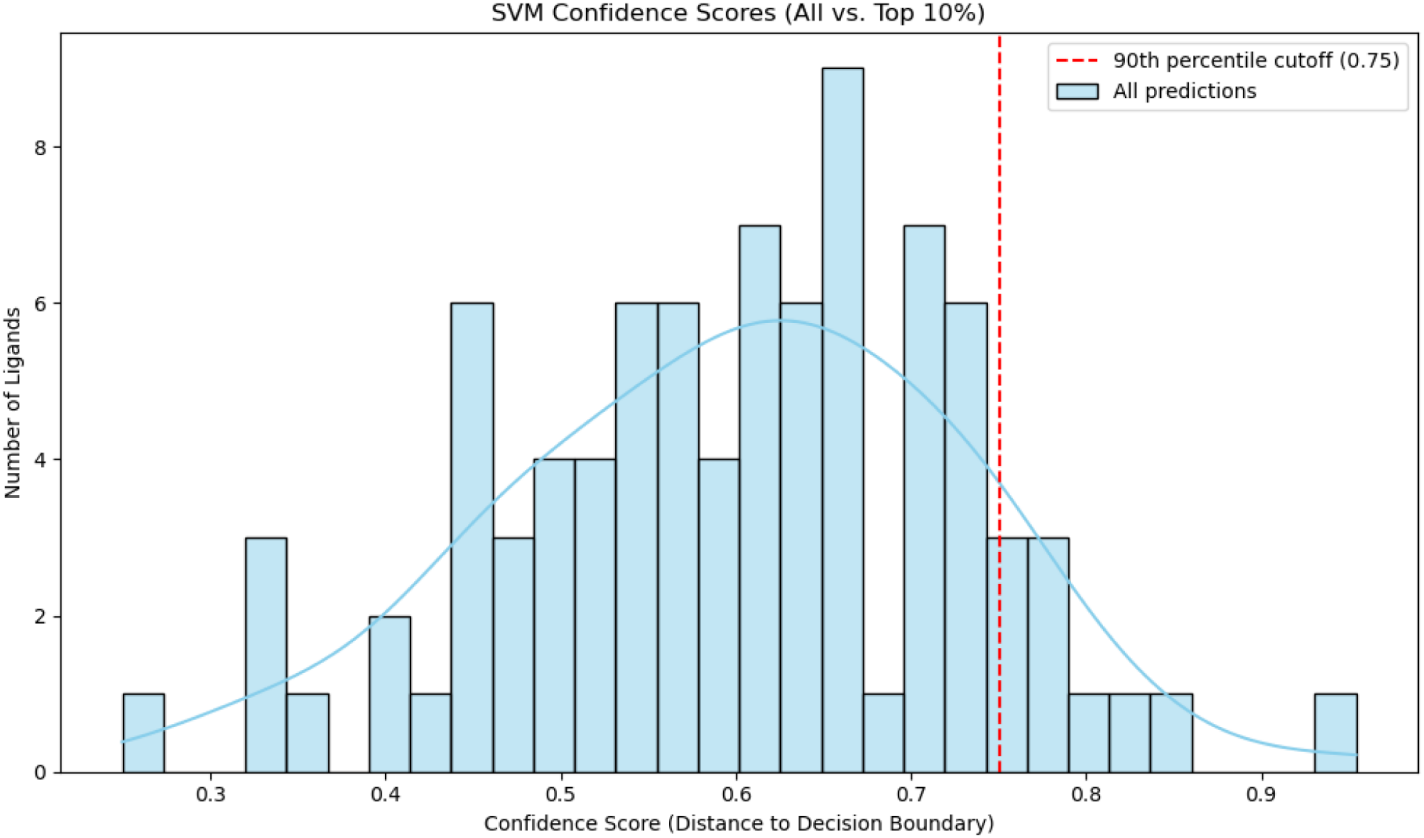
Distribution of SVM confidence scores for 87 predicted HCAR1 antagonists. All 87 compounds were classified as antagonists (label = 1) by the support vector machine (SVM) model. The histogram shows the distribution of confidence scores, measured as the distance from the decision boundary. Most ligands exhibited moderate confidence, centered around a score of ∼0.65. The red dashed line indicates the 90th percentile cutoff (score = 0.75), above which the top 10% most confidently predicted antagonists (*n* = 9) were selected for prioritization.

**Table 3:** Top 10% Most Confidently Predicted Antagonists Targeting HCAR1, Identified from SVM Classification of 87 Candidate Compounds

Ligand ID	Δ*G* active	Δ*G* inactive	ΔAffinity	Label	Confidence Score	Confidence Percentile
CID 3822	–6.861	–9.387	2.526	Antagonist	0.95	1.0
NuBBE 580	–6.092	–8.859	2.767	Antagonist	0.76	0.92
CID 5479529	–4.9	–8.122	3.222	Antagonist	0.75	0.91
NuBBE 582	–5.877	–8.734	2.857	Antagonist	0.82	0.98
NuBBE 1572	–6.138	–9.217	3.079	Antagonist	0.77	0.93
CID 6758/NuBBE 1414	–5.715	–9.208	3.493	Antagonist	0.85	0.99
NuBBE 1145	–5.591	–10.072	4.481	Antagonist	0.77	0.94
NuBBE 1106	–7.44	–9.705	2.265	Antagonist	0.79	0.95
CID 31703	–4.565	–9.749	5.184	Antagonist	0.79	0.96

### 3.5 Prioritization of High-Confidence Antagonist Candidates

Molecular docking simulations conducted between the top 10% high-confidence ligands and the Class A GPCRs used in model training resulted in the prioritization of ligands with the lowest Off-Target Scores and positive HCAR1 ΔAffinity. The top candidates, listed in Table 4, include Ketanserin (CID 3822), Cryptopyranmoscatone A1 diacetate (NuBBE 580), and Cefuroxime (CID 5479529), which showed ΔAffinity values of 2.5–3.2 for HCAR1 and minimal off-target interaction potential (Off-Target Scores between 12.8 and 14.6).

**Table 4:** Top-Prioritized Ligands Based on Off-Target Assessment and HCAR1 Selectivity

Compound ID	Name	ΔAffinity (HCAR1)	Off-Target Score
CID 3822	Ketanserin	2.526	12.807
NuBBE 580	Cryptopyranmoscatone A1 diacetate	2.767	13.658
CID 5479529	Cefuroxime	3.222	14.597
NuBBE 582	Cryptopyranmoscatone B1 diacetate	2.857	19.945
NuBBE 1572	Aricine	3.079	21.743
CID 6758/NuBBE 1414	Rotenone	3.493	23.525
NuBBE 1145	20,21,22,23-tetrahydro-23-oxoazadirone	4.481	29.663
NuBBE 1106	3-(2-(7,7-dimethyl-3,7-dihydropyrano[3,2-e]indol-1-yl)ethyl)-1-hydroxyquinazoline-2,4(1H,3H)-dione	2.265	34.569
CID 31703	Doxorubicin	5.184	42.194

As shown in the phylogenetic dendrogram (Figure 1), HCAR1 belongs to the same Class A GPCR subcluster as HCAR2, HCAR3, GPR35, and OXER1. Additionally, SUCNR1 shares a significant ligand-type similarity (both succinate and lactate are small carboxylic acids). Accounting for the conservation of structural features relevant to ligand recognition, we provide the ΔAffinity values against HCAR2, HCAR3, OXER1, GPR35, and SUCNR1 in Table S6.

### 3.6 Off-Target Risk and Selectivity Landscape

One hundred thirteen informative fingerprint bits were featured from the top 10% most confidently predicted antagonists (9 ligands). Among them, 20 bits had the strongest monotonic relationship with OffTarget_Score. However, two bits (bits 1060 and 80) could not be mapped to substructures and were therefore excluded. The remaining 18 top bits/substructures revealed two distinct trends: (i) Positive correlated substructures associated with off-target binding, and (ii) Negative correlated substructures associated with receptor-specific binding (Figure 5).

**Figure 5:**
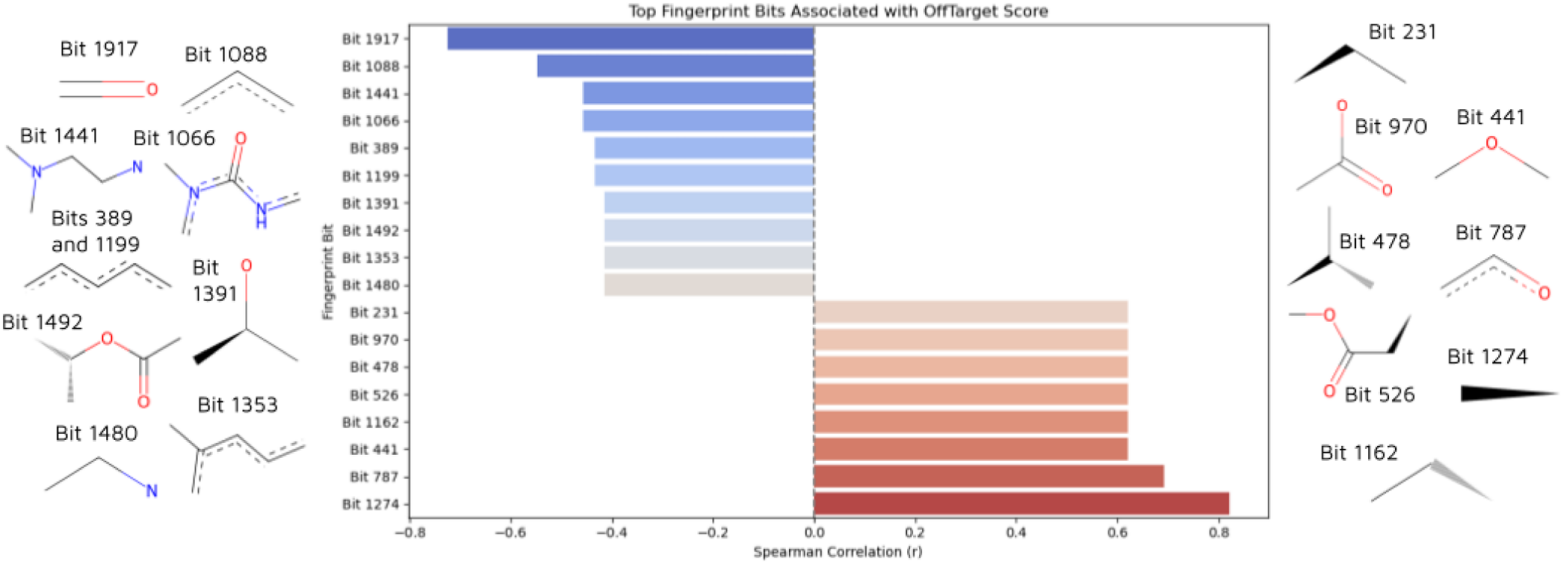
Top fingerprint bits correlated with Off-Target Score and their representative substructures. Eighteen structurally mapped bits from the top 10% most confidently predicted antagonists showed distinct trends. Positively correlated bits (right, red) were mostly nonpolar and aliphatic (e.g., ethyl, propyl, ester groups), associated with a higher off-target risk. Negatively correlated bits (left, blue) included polar and aromatic features (e.g., carbonyl, substituted aryls), suggesting receptor-specific binding.

Positively correlated substructures were generally nonpolar and aliphatic, being represented by ethyl (bit 1247), propyl (bits 1162 and 231), isobutyl (bit 478), and ester functionalities (bit 526). On the other hand, polar, rigid, and aromatic features, such as carbonyl (bit 1917), aromatic rings (bits 1088, 1199, and 389), as well as functionalized motifs like bit 1492, may support receptor-specific binding, suggested by Spearman correlation coefficients below −0.4.

### 3.7 Binding Mode Analysis

All three compounds established strong interactions with key residues within the orthosteric pocket of the HCAR1-modeled inactive conformer (most notably Arg240 and Tyr268). Mutagenesis studies have shown that substituting either residue with alanine (R240A or Y268A) significantly reduces HCAR1 activation by the selective agonist 3,5-DHBA.^20^ Notably, all ligands formed tight polar contacts with Arg240 (Figures S1–S3), a residue whose upward displacement is important for receptor activation. Thus, by stabilizing Arg240 in a downward, inactive-state position, these ligands likely hinder the conformational transition required for activation, reinforcing their predicted antagonist behavior.

Interestingly, none of the compounds formed salt bridges or close polar contacts with Arg99 (3.36), a residue often implicated in Gi-coupling, whose substitution abrogates HCAR1 activation. This absence was consistent across both the active and inactive conformers, suggesting a lack of interaction with regions associated with intracellular signaling, another feature consistent with functional antagonism.

Additionally, the compounds formed multiple hydrophobic and polar interactions that contribute to tight binding in the inactive state. As shown in Figure S1A,B, Ketanserin (CID 3822) features extended benzoic groups that are deeply packed and stabilized by Leu115 (TM3) and Ala148 (TM4). These interactions enhance conformational locking of the ligand in the core of the receptor.

In contrast, docking to the active conformers resulted in fewer and more peripheral polar contacts (Figures S1–S3C,D), often located near the extracellular loop 2 (ECL2) rather than within the orthosteric site. The ligands appeared less deeply buried and less stabilized, which is consistent with their higher binding free energies to the active state (>2.5 kcal/mol) and supports the proposed selectivity for the inactive conformation.

### 3.8 Model Validation on Known HCAR1 Agonists

As shown in Table 5, both the internal and external validation sets of HCAR1 agonists were correctly classified with high probability (>85%) and high confidence scores (>0.85). This result implies that the model is stable, reproducible, and ultimately accurate in predicting the target’s known ligands. At first glance, it may appear that the model would inevitably correctly classify the internal set by having “memorized” it. However, SVM models’ decision boundaries rely on a weighted combination of all features of the training set, not individual molecules.^52^ Overall, these results indicate that the model behaves consistently after being saved and reloaded (internal validation) and, more importantly, can generalize to unseen data (external validation).

**Table 5:** SVM Prediction Results for IUPHAR-Listed HCAR1 Agonists

Name	Validation set	CID	Active	Inative	Predicted class	Predicted probability (%)	Confidence score
GPR81 agonist 1	Internal	86279608	–6.140	–8.986	Agonist	96.9	0.857
3,5-Dihydroxybenzoic Acid	Internal	7424	–6.452	–5.240	Agonist	95.9	0.959
D-Lactic Acid	External	61503	–4.255	–4.091	Agonist	95.7	0.957
L-Lactic Acid	Internal	107689	–4.086	–4.071	Agonist	95.7	0.967
4-Hydroxybutanoate	External	0370332	–4.186	–4.248	Agonist	87.5	0.875
Nicotinic Acid	External	938	–5.304	–4.643	Agonist	95.8	0.958

### 3.9 Impact of Antagonist-Driving Fragments on Binding-Site Geometry and Ligand Solvent Accessibility

Before analyzing ligand-specific effects, we evaluated whether the active and inactive HCAR1 conformers differed intrinsically in their pocket geometry. Using DogSiteScorer, the inactive state showed a noticeably larger internal pocket, with greater volume, surface area, and depth than the active conformer (Table S7). This broader cavity is consistent with the expected openness of GPCR inactive states. It provides a structural basis for the improved accommodation of bulkier substituents, such as the isopropenyl ether and methyl-ketone groups examined below.

The visual inspection of the molecular docking complexes of the analogs’ best-scoring poses with the active conformation of HCAR1 showed that the presence of the antagonist-driving fragments did not significantly alter the overall stabilization pocket, with all remaining within the binding site, as shown in Figures 6 and 7D–F.^20^ This effect was particularly evident for γ-hydroxybutyrate and its analogs, where all poses clustered below the extracellular loop (ECL), maintaining key contacts with R71, L264, and H261 (Figure 7D–F). Interestingly, the isopropenyl ether moiety established extra van der Waals interactions with TM3 residues (L92, L95, and R99), suggesting that it fits neatly within an extended region of the binding pocket. This behavior is consistent with a restriction-lock effect, where bulky substituents limit pocket closure (Figure 7F). This interpretation aligns with the higher burial depth of the isopropenyl ether (ΔSASA = 145 Å^2^) compared with the methyl-ketone analog (ΔSASA = 133 Å^2^) and the native ligand (ΔSASA = 107 Å^2^), as shown in Table S8.

**Figure 6:**
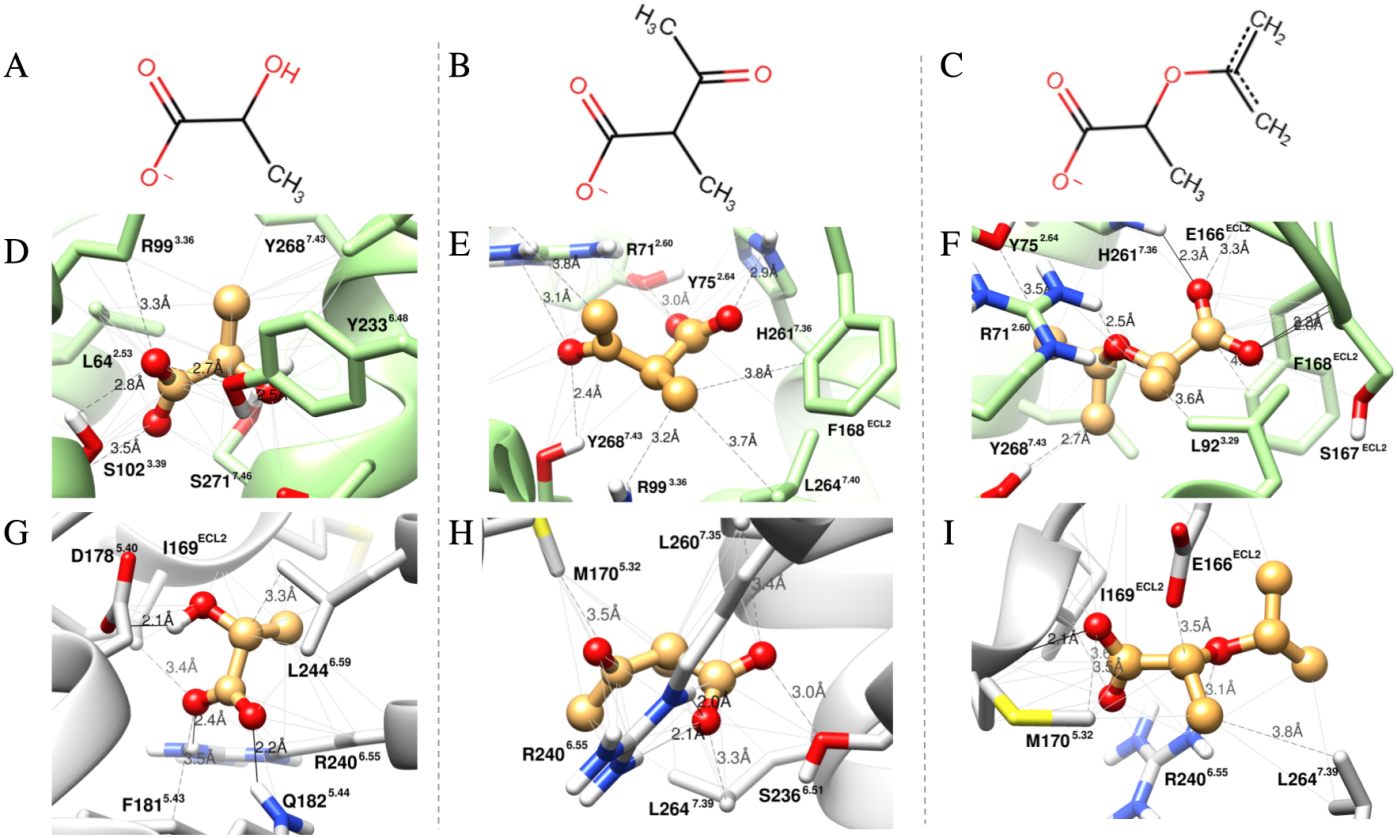
Predicted binding modes of lactate and its analogs, 2-acetylpropanoate and 2-(prop-1-en-2-yloxy)­propanoate in the active (D–F) and inactive (G–I) conformations of HCAR1. (A–C) Two-dimensional representations of the respective ligands. Dashed lines represent van der Waals interactions, and bold lines represent hydrogen bonds computed in UCSF Chimera 1.16. (D) In the active conformation (PDB 9IZD), the best docking pose of lactate lies below the backbone of Arg99^3.36^, within the toggle-switch motif, forming a hydrogen bond (2.5 Å) with Tyr233^6.48^. (E–F) The introduction of bulkier substituents—particularly the isopropenyl ether group—appears to shift the ligand upward toward the extracellular region (ECL2; Phe168 and Glu166), increasing contacts with peripheral pocket residues, including Leu92^3.29^, Phe168, and Leu264^7.39^. (G–I) In the inactive conformation modeled from HCAR2 (PDB 7ZLY), all ligands occupy a similar hydrophobic cavity near Arg240^6.55^, Met170^5.23^, and Leu260^7.35^. Created by the authors using UCSF Chimera 1.16 and Marvin JS (ChemAxon). Ligand structures were drawn in Marvin JS, and molecular visualizations were generated in UCSF Chimera.

**Figure 7:**
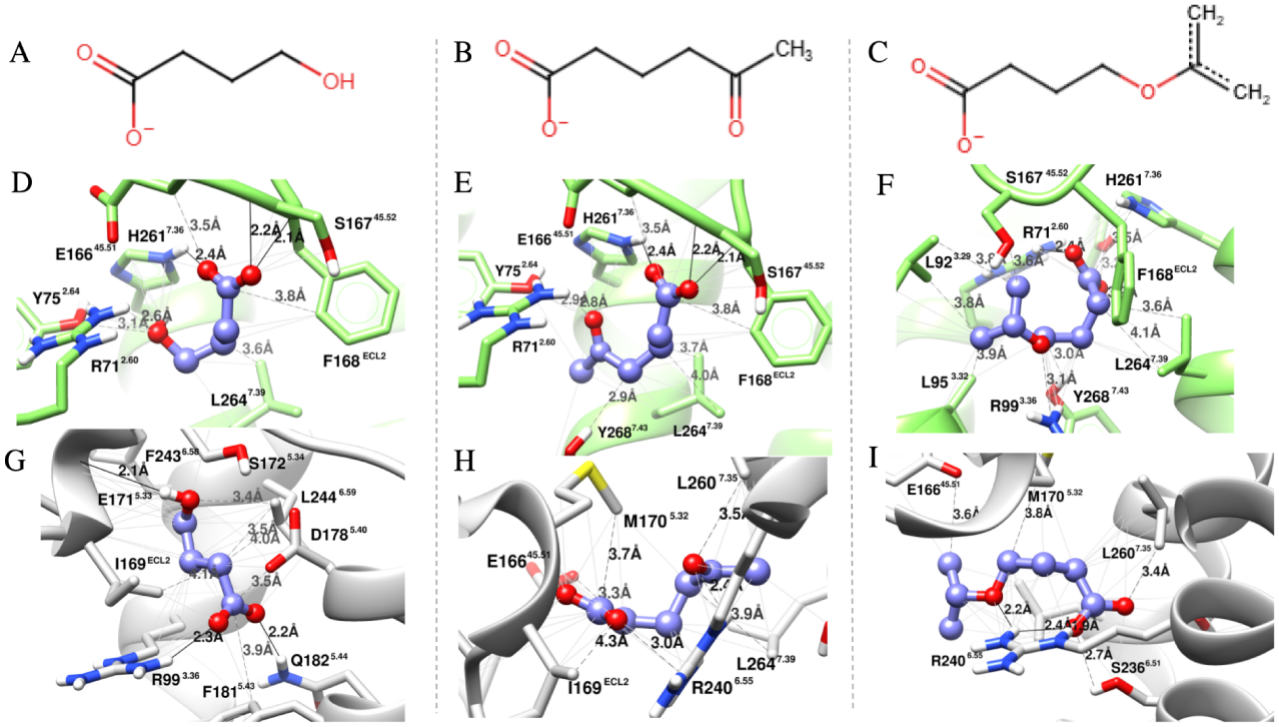
Predicted binding modes of γ-hydroxybutyrate and its analogs 5-acetylpentanoate and 4-(prop-1-en-2-yloxy)­butanoate in the active (D–F) and inactive (G–I) conformations of HCAR1. (A–C) Two-dimensional representations of the respective ligands. Dashed lines represent van der Waals interactions, and bold lines represent hydrogen bonds computed in UCSF Chimera 1.16. (D) In the active conformation (PDB 9IZD), interact with Arg71^2^·^60^, Tyr75^2^·^64^, His261^7^·^36^ and Ser167^45^·^52^. (E–F) The introduction of bulkier substituents, particularly the isopropenyl ether group, facilitates interactions with hydrophobic residues such as Leu92^3^·^29^, Leu95^3^·^23^, and Leu264^7^·^39^, (G–I) In the inactive conformation, γ-hydroxybutyrate’s best pose forms a hydrogen bond with the crucial binding residue Arg99^3^·^36^. At the same time, the analogs appear pushed toward the upper region, forming additional contacts with Arg240^6^·^55^, Met170^5^·^23^, and Leu260^7.35^. Created by the authors using UCSF Chimera 1.16 and Marvin JS (ChemAxon). Ligand structures were drawn in Marvin JS, and molecular visualizations were generated in UCSF Chimera.

As illustrated in Figures 6 and 7G–I, in the inactive conformation, both lactate and γ-hydroxybutyrate analogs followed a similar interactional pattern involving TM5 (M170), TM6 (R240), and TM7 (L264), with only minor shifts. The exception was native γ-hydroxybutyrate, which formed hydrogen bonds with internal residues R99 (3.36) and Q182 (5.44) (Figure 7G). The differential SASA between conformations was negligible for lactate and its analogs, so our analysis focused on γ-hydroxybutyrate, where the contrast was clearer (Figure S6).

Curiously, γ-hydroxybutyrate, an agonist expected to stabilize the active conformation preferentially, showed a greater pocket burial in the inactive conformation (Differential SASA = 2.520). Adding the methyl-ketone shifted the active conformer stabilization to a more favorable value (Differential SASA = −1.626), whereas the presence of isopropenyl ether seems to have increased the ligand accommodation in the inactive conformation, reinforcing the hypothesis of restriction lock (Table S8).

Naturally, working with rigid receptor models imposes clear limitations, particularly when applying SASA, which is highly sensitive to induced-fit effects and more reliable when coupled with molecular dynamics simulations.^54^ Because the inactive conformation has a larger internal volume, polar and bulky ligands, such as γ-hydroxybutyrate, may appear more deeply buried, regardless of their activity. Still, this does not compromise the relative comparison among analogs and their native ligands, which helps minimize model-dependent artifacts. SASA, therefore, should be seen as a relative indicator—a geometric complement to docking scores that helps overcome the limitations of scoring functions treating solvent implicitly.^31^

Altogether, the interaction patterns and SASA results suggest that the isopropenyl ether fragment—highlighted by the SVM model and SHAP analysis as a likely driver of antagonism—could exert this influence when replacing the hydroxyl group in bulkier ligands, such as γ-hydroxybutyrate. Chemically, the isopropenyl ether group is mildly electron-donating and partially conjugated, introducing both polarity and steric bulk to the ligand surface. These characteristics can subtly influence nearby hydrophobic residues, allowing the group to act as a rigid hydrophobic anchor that facilitates the ligand’s settlement within the pocket. As shown in Figure 7F, this moiety forms close contacts with L92, L95, R99, and Ser167.

### 3.10 SVM Classification of Fragment-Modified Ligands

As shown in Table 6, all analogs were still classified as agonists, which is consistent with their templates (Table 5). Interestingly, the γ-hydroxybutyrate analog carrying the isopropenyl ether group showed a drop in both predicted probability and confidence score (≈0.67), reflecting a lower certainty in agonist classification, which aligns with the postdocking solvent accessibility analysis for this analog (Section 3.8). This behavior was not observed for the remaining analogs, suggesting that, despite the highly influential power of the substructures, the model captures the overall molecular context of the query compounds rather than the unique features. Nonetheless, we anticipate that the same values observed for the predicted probability and confidence score may cast doubt on the power of these metrics. In fact, it may arise from the SVM’s calibrated decision function, where a stable margin between classes leads both metrics to converge naturally.^55^

**Table 6:** SVM Prediction Results for Lactate and the γ-Hydroxybutyrate Designed Analogs

Chemical name (IUPAC)	Active	Inative	Predicted class	Predicted probability (%)	Confidence score
2-hydroxypropanoate (lactate)	–4.300	–3.727	Agonist	85.5	0.855
prop-1-en-2-yl)oxy]propanoate	–4.850	–4.414	Agonist	84.4	0.844
2-acetylpropanoate	–4.959	–4.503	Agonist	84.3	0.843
4-hydroxybutanoate (y-hydroxybutyrate)	–4.201	–4.267	Agonist	87.7	0.877
5-acetylpentanoate	–5.212	–4.515	Agonist	85.9	0.859
4-(prop-1-en-2-yloxy)butanoate	–4.921	–4.557	Agonist	67.7	0.677

### 3.11 Rational Framework for the Design of GPR81 Antagonists

In medicinal chemistry, the design of competitive antagonists typically starts from the endogenous agonist scaffold. It aims to mimic the agonist binding mode while avoiding receptor activation (bioisosteric replacements), which implies the need for insertion of heavy groups that favor conformational restriction.^56^ In general, antagonists tend to exhibit a higher molecular weight, often accompanied by moderate lipophilicity.^57^ This well-known fact was captured by our SVM model, where MWs closer to 500 had the highest impact on antagonist prediction (Figure 3A) among the physicochemical descriptors evaluated.

In the context of our emerging target, HCAR1, its prototypical endogenous ligand, L-lactate, is a relatively simple molecule that could be summarized in chemical and functional terms as assembled by three groups (Figure 8A): 1) an acidic carboxylate group (negatively charged), which forms a salt bridge with the positively charged arginine residue R99 that induces its 90° rotation essential for receptor activation; 2) the nonacidic hydroxy group, that provides additional polar interactions; 3) the methyl group, a hydrophobic anchor. This knowledge appears sufficient to provide adequate replacements, as numerous bioisosters are available for those groups. However, this apparent advantage has a more intricate and nuanced background: bioisosteric replacements are context-dependent.^58^

**Figure 8:**
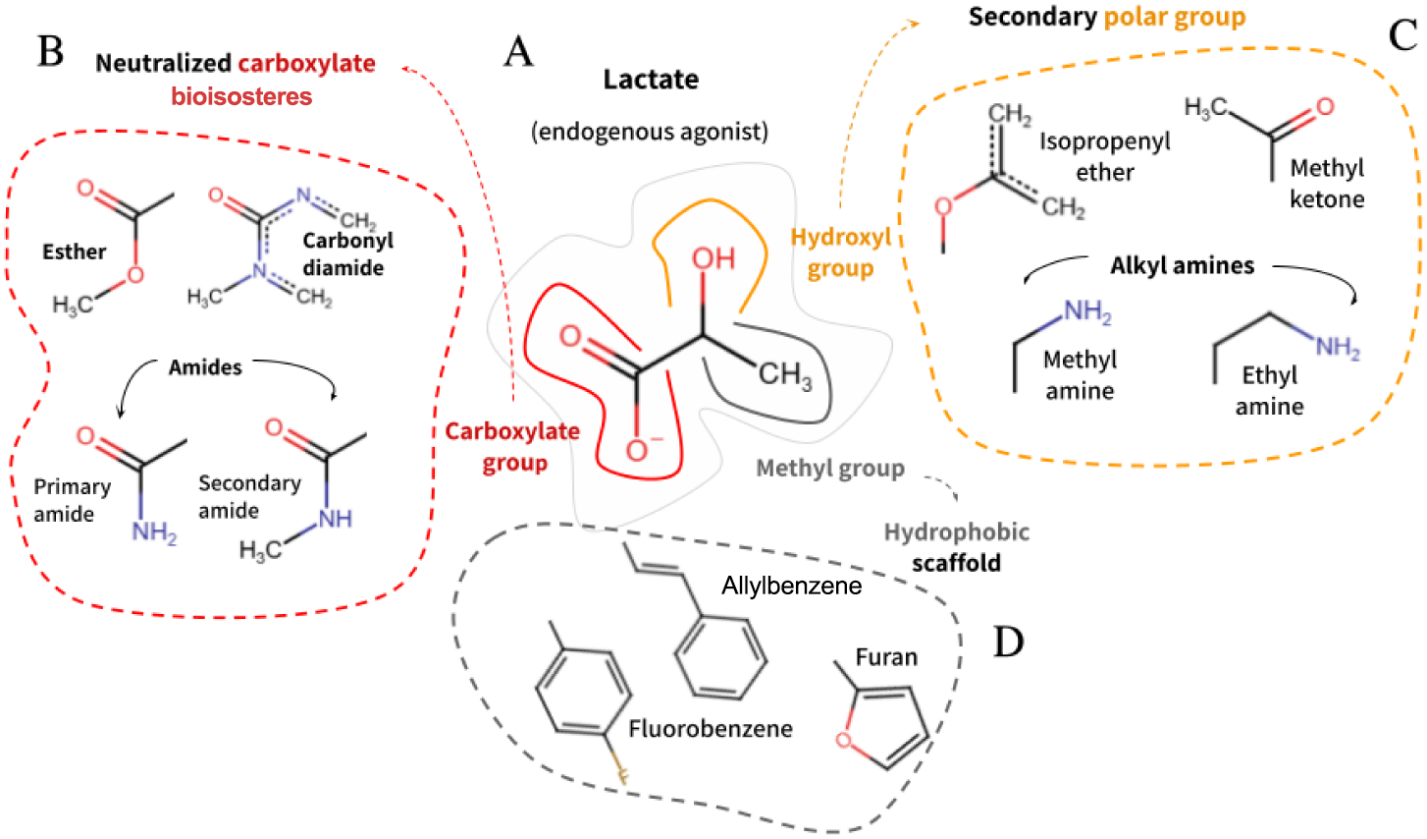
Structure-guided rationale for GPR81 antagonist design. Schematic representation of the bioisosteric replacement strategy derived from the physicochemical and fragment-based insights of the SVM model combined with ensemble docking analyses. (A) The endogenous agonist L-lactate served as the reference scaffold, featuring a carboxylate and hydroxyl groups, as well as a methyl anchor. (B) Neutralized carboxylate bioisosteres—including esters, amides, and carbonyl diamides—maintain the carboxylate geometry while uncharged. (C) Hydroxyl substitutions can lower polarity and improve electronic tunability. Methyl ketone (bit 370), isopropenyl ether (bit 249), and alkyl amines can modulate local electrostatics, thereby influencing the acidity of the carboxylate group. (D) Extension of the methyl anchor with aromatic or bulky hydrophobic groups, such as fluorobenzene, allylbenzene, or furan, may strengthen apolar packing and favor stabilization of the inactive receptor state.

Our combined SVM and ensemble docking analyses, supported by known molecular entities (Figures S1–S3), allowed us to outline a preliminary replacement rationale (Figure 8). First, neutralizing the carboxylate group while maintaining its planar geometry may impair the salt bridge interaction, providing a similar packing of the carboxylate moiety with lower bonding strength. As such, and in correspondence with leading low-off-target substructures (Figure 5), ether groups (bits 1917 and 1492), amides (bits 1917, 1441, 1066, and 1480), and carbonylamides (bits 389, 1199, and 1391) emerge. The hydrogen bonds established with R240 by the carbonyl oxygen of the carbonyl diamine and the cyclic amide of the β-lactam ring in Ketanserin (Figure S1) and Cefuroxime (Figure S3), respectively, illustrate that.

We also propose that replacing the hydroxyl group with less polar fragments can fine-tune the local electronic environment. Among these, the methyl ketone (bit 370), isopropenyl ether (bit 249), and alkyl amines highlighted by the SHAP analysis (Figure 3B–C) stand out (Figures 7 and S2).

The methyl ketone, a mildly electron-withdrawing group, may increase the acidity of the nearby carboxylate and strengthen polar interactions—an effect compatible with agonist-like behavior. When introduced together with an amide or carbonylamide replacement (Figure 8B), however, the charge distribution may change, softening these polar contacts and producing a more neutral environment within the pocket. In contrast, the isopropenyl ether acts as a gentle electron donor, slightly lowering carboxylate acidity and enhancing hydrophobic character—traits commonly associated with antagonist stabilization. Finally, the alkyl amines, classical bioisosteres of hydroxyl groups, introduce a basic, protonatable center that may reduce overall polarity while still allowing alternative hydrogen-bonding arrangements within the receptor cavity.

Lastly, extending the methyl anchor with bulkier or aromatic groups may restrict the allosteric movements required for receptor activation. The inactive HCAR1 conformer molecular docking complexes with Ketanserin (Figure S1), Cryptopyranmoscatone A1 diacetate (Figure S2), and Cefuroxime (Figure S3) illustrate this through the packing of fluorobenzene, allylbenzene, and furan rings, which strengthen apolar contacts with Leu150, Ala96, and Leu264. Notably, the fluoro-substituted alkene (bit 497), being one of the top antagonist-driving fragments identified by SHAP, reinforces the idea that introducing polarizable, hydrophobic scaffolds can favor the stabilization of inactive states and contribute to antagonism.

## 4 Discussion

Recently, the FDA-approved antihypertensive drug reserpine emerged as a potential antagonist of HCAR1 by impairing its lactate-induced activation in mice harboring immunotherapy-resistant colorectal cancer.^8^ As a result, the antitumor immune response (e.g., CD8+ T cell activity) was restored, along with responsiveness to anti-PD-1 antibody therapy. Interestingly, the desired molecular patterns proposed here for HCAR1 potential antagonists are highly present in reserpine’s chemical structure.

The electron-donating aromatic fragment highlighted in our model—previously represented by an isopropenyl ether-like pattern—is functionally captured in reserpine by the 3,4,5-trimethoxybenzoyl group (TMBA), which provides a similar electron-rich environment.^59^ Similarly, the neutral ester-type carboxylate bioisostere appears twice: as a linkage between TMBA and the reserpic acid moiety, and in the yohimban skeleton, the five-fused-ring indole alkaloid system.^59^

Reserpine has a high molecular weight, primarily due to its bulky nature. This structural trend is crucial for the irreversible inhibition of its therapeutic target VMAT2, particularly due to deep occlusion and steric locking within a high-volume binding pocket.^60^ Inactive states of GPCRs, including HCAR1, often present a more open conformation with greater binding-site volume,^61^ which may favor the occupancy of ligands such as reserpine. Consistently, our top three potential antagonists show a similar tendency, delving deeply into the orthosteric pocket of the inactive conformer.

Ketanserin, a 5-HT2A antagonist and one of our leading compounds, was also resolved bound to human VMAT2 (PDB ID 8JTB).^60^ In that structure, it occupies a deep lumen-facing pocket in a manner reminiscent of reserpine’s high-volume engagement. It supports the suitability of bulky, heteroatom-rich scaffolds for stabilizing expanded receptor pockets and aligns with ketanserin’s strong ΔAffinity (inactive–active) and its deep orthosteric engagement in our HCAR1 models.

Reserpine’s natural origin further highlights another promising source of antagonist-like scaffolds. It is an indole alkaloid from *Rauvolfia serpentina* (*Apocynaceae*), the same family as the Vinca alkaloid-producing *Catharanthus roseus*.^62^ Although structurally distinct, the*Cryptocarya moschata* compound prioritized in this work, Cryptopyranmoscatone A1 diacetate, shares relevant patterns: a multiring system with electron-donating groups and two aliphatic ester units.^63,64^

Ultimately, the alignment between the experimental evidence and antagonist-driven features elucidated here highlights the translational strength of our integrated pipeline. Naturally, functional studies will be required to confirm antagonism—a requirement in any early-stage discovery workflow. Even so, the present work defines a clear, chemically plausible antagonist space for HCAR1, offering concrete, experimentally tractable candidates for subsequent validation.

## 5 Conclusion

This work provides a mechanistically grounded initial framework for the early-stage drug discovery of HCAR1 antagonists, leveraging accessible tools. By docking with an interpretable SVM model, we were able to narrow down a broad compound set to a few molecules that consistently exhibited features expected of an antagonist. Some of these, such as Cryptopyranmoscatone A1 diacetate and Ketanserin, exhibit molecular patterns—bulky groups and electron-donating fragments—that align with current knowledge derived from recent experimental data. These observations help outline the “antagonist space” of HCAR1. They also point to compounds that can be tested next. Altogether, the results give a starting point for fragment refinement and experimental follow-up, especially for cancers where lactate signaling plays a central role.

## Supplementary Material



## Notes

Supplementary figures (Figures S1–S6); supplementary tables (Tables S1–S8); homology modeling metrics; docking grid parameters; off-target affinity matrices; and solvent-accessible surface area (SASA) analyses (PDF)

## Abbreviations

ADORA2A: Adenosine A_2A_ receptor
ADRA1A: Adrenergic α_1A_ receptor
AGTR1: Angiotensin II type 1 receptor
AUC: Area Under the Receiver Operating Characteristic Curve
CHBA: 3-chloro-5-hydroxybenzoic acid
ECFP4: Extended-Connectivity Fingerprint, radius 2
ECL2: Extracellular Loop 2
FDA: Food and Drug Administration
FBDD: Fragment-Based Drug Design
GPCR: G protein-coupled receptor
GPR35: G protein-coupled receptor 35
HCAR1: Hydroxycarboxylic acid receptor 1 (GPR81)
HCAR2: Hydroxycarboxylic acid receptor 2 (GPR109A)
HCAR3: Hydroxycarboxylic acid receptor 3 (GPR109B)
IUPHAR: International Union of Basic and Clinical Pharmacology
MCHR1: Melanin-concentrating hormone receptor 1
MW: Molecular Weight
NuBBE: Núcleo de Bioensaios, Biossíntese e Ecofisiologia de Produtos Naturais
OPRD1: Delta opioid receptor
OXER1: Oxoeicosanoid receptor 1
P2RY2: Purinergic receptor P2Y2
PDB: Protein Data Bank
PDAC: Pancreatic ductal adenocarcinoma
RBF: Radial Basis Function
ROC: Receiver Operating Characteristic
SHAP: SHapley Additive exPlanations
SMILES: Simplified Molecular Input Line Entry System
SUCNR1: Succinate receptor 1
SVM: Support Vector Machine
TPSA: Topological Polar Surface Area
ΔAffinity: Difference in docking affinity between active and inactive receptor conformers
