# Supplementary material for: A Machine Learning-Guided Approach for Identifying Potential HCAR1 Antagonists in Lactate-Driven Cancers
Source: ACS Omega. 2026 Feb 3;11(6):9354–68. doi: 10.1021/acsomega.5c09253 (PMC12917714; doi:10.1021/acsomega.5c09253)

Supporting Information

A Machine Learning-Guided Approach for Identifying Potential HCAR1 Antagonists in Lactate-Driven Cancers

Letícia Vivas Carvalho[1], Núbia Seyffert[1], Roberto Meyer[1], Sandeep Tiwari[1], Thiago Luiz de Paula Castro[1,2]

[1] Institute of Health Sciences (ICS), Federal University of Bahia (UFBA), Av. Reitor Miguel Calmon, s/n, Canela, 40231-300, Salvador, Bahia, Brazil.

[2] Institute of Biological Sciences (ICB), Federal University of Minas Gerais (UFMG), Av. Presidente Antônio Carlos, 6627, Pampulha, 31270-901, Belo Horizonte, Minas Gerais, Brazil.

Table S1. Query sequences used for homology modeling and structural analysis. For each GPCR included in the dataset, the table lists the human UniProt identifier, organism of origin, and NCBI taxonomic identifier used as input for sequence alignment and template selection.

Name	Query sequence (Uniprot ID)	Organism	Taxonomic identifier (NCBI)
HCAR1	Q9BXC0	*Homo sapiens*	9606
HCAR3	P49019	*Homo sapiens*	9606
OXER1	Q8TDS5	*Homo sapiens*	9606
GPR35	Q9HC97	*Homo sapiens*	9606
P2RY2	P41231	*Homo sapiens*	9606
AGTR1	P30556	*Homo sapiens*	9606
OPRD1	P41143	*Homo sapiens*	9606

Table S2. Structural templates and quality metrics for selected GPCRs. The table lists the UniProt ID, PDB ID, resolution (in Å), conformational state (active or inactive), and bound ligand for each receptor.

Receptor	Uniprot ID	PDB ID	Resolution (Å)	Conformation	Ligand
HCAR1	Q9BXC0	9IZD	3.16	Active	CHBA
HCAR2	Q8TDS4	8H2G	3.01	Active	Niacin
HCAR2	Q8TDS4	7ZL9	2.70	Inactive	—-
HCAR3	P49019	8JEF	2.96	Active	3HO
GPR35	Q9HC97	8H8J	3.20	Active	Lodoxamide
AGTR1	P30556	7F6G	2.90	Active	Sar1-AngII
SUCR1	Q9BXA5	8WOG	2.97	Active	Succinate
SUCR1	Q9BXA5	6Z10	2.27	Inactive	Q4T
OPRD1	P41143	8Y45	3.45	Active	A1LXY
MCHR1	Q99705	8WSS	3.01	Active	Pro-MCH
MCHR1	Q99705	8YNS	3.33	Inactive	SNAP-94847
ADRA1A	P35348	7YM8	2.92	Active	oxymetazoline
ADRA1A	P35348	7YMJ	3.35	Inactive	Tamsulosin
ADORA2A	P08913	2YDV	2.60	Active	NECA
ADORA2A	P08913	4EIY	1.80	Inactive	ZM241385

Table S3. Homology modeling templates and quality metrics. Templates with greater than 25% sequence identity were used in the absence of available receptor conformers. Only models with ≥85% favored Ramachandran residues were accepted. RMSD values reflect the superimposition of the model to the template.

Receptor	Template PDB ID	Conformation	Sequence Identity (%)	Ramachandran Favored (%)	RMSD (Å)
HCAR1	7ZL9	Inactive	52.49	96.6	0.211
HCAR3	7ZL9	Inactive	95.30	92.9	0.145
OXER1	8JEF	Active	40.82	93.0	0.092
OXER1	7ZL9	Inactive	39.80	89.6	0.130
GPR35	7ZL9	Inactive	32.69	93.9	0.267
P2RY2	7XXH	Active	34.93	92.5	0.115
P2RY2	6Z10	Inactive	32.36	90.9	0.223
AGTR1	7JNI	Inactive	37.94	93.8	1.234
OPRD1	9MQH	Inactive	62.71	94.6	0.193

Table S4. Grid parameters for active receptor conformers. Listed are the receptor, structure or model ID, grid center coordinates (X, Y, Z), grid size, and centering method.

Receptor	PDB ID/Model	Model Grid Center (X, Y, Z)	Grid Size (Å³)	Center-Based On
HCAR1	9IZD	128.6, 144.4, 95.6	25	Co-crystallized ligand (CHBA)
HCAR3	8JEF	132.2, 127.5, 172.4	25	Co-crystallized ligand (3HO)
HCAR2	8H2G	119.6, 114.2, 161.7	25	Co-crystallized ligand (Niacin)
OXER1	Homology modeling	130.5, 126.2, 174	25	R137, Y306, L302
GPR35	8H8J	144, 141, 162	25	Co-crystallized ligand (Lodoxamide)
SUCR1	8WOG	133.9, 142.3, 101.1	25	Co-crystallized ligand (Succinate)
OPRD1	8Y45	111.5, 117.3, 138.9	25	Co-crystallized ligand (A1LXY)
MCHR1	8WSS	111.5, 104.7, 137.8	25	Co-crystallized ligand (MCH)
ADA1	7YM8	138.2, 141.8, 147.6	25	Co-crystallized ligand (oxymetazoline)

P2RY2	Homology modeling	154, 111.2, 144.3	25	R110, R265, R292
AGTR1	7F6G	118.6, 129.4, 105.5	25	Co-crystallized ligand (Sar1-AngII)
ADORA2A	2YDV	30.3, 10.2, -17.5	25	Co-crystallized ligand (NECA)

Table S5. Grid parameters for inactive receptor conformers. Listed are the receptor, structure or model ID, grid center coordinates (X, Y, Z), grid size, and centering method.

Receptor	PDB ID/Model	Model Grid Center (X, Y, Z)	Grid Size (Å^3)	Center-Based On
HCAR1	Homology modeling	20.8, 72.8, 191.4	25	R71, R99, R240, Y268
HCAR3	Homology modeling	18.7, 72.5, 191	25	R111, R251, Y284
HCAR2	7ZL9	19.4, 68.2, 192.1	25	R111, R251, Y284
OXER1	Homology modeling	130.5, 126.2, 174	25	R87, Y112, Y116
GPR35	Homology modeling	22, 71.1, 188.7	25	R100, Y96, R151, R164
SUCR1	6Z10	-4.6, 10.48, 17.18	25	Co-crystallized ligand (Q4T)
OPRD1	Homology modeling	154.6, 152.7, 190.8	25	D95, W284, V296, V296
MCHR1	8YNS	134.8, 133.7, 105.2	25	Co-crystallized ligand (SNAP-94847)
ADA1	7YMJ	107.3, 113.9, 132.3	25	Co-crystallized ligand (tamsulosin)
P2RY2	Homology modeling	-4.44, 10.5, 20.7	25	R110, R265, R292

AGTR1	Homology modeling	-4.9, 10.38, 18.7	25	R167, W253,Y351
ADORA2A	4EIY	-0.87, 6.27, 21.4	25	Co-crystallized ligand (ZM241385)

Table S6. ΔAffinity values (kcal/mol) for top-predicted HCAR1 antagonists across phylogenetically related Class A GPCRs (as shown in Figure 1).

Ligand ID/Name	ΔAffinity (HCAR2)	ΔAffinity (HCAR3)	ΔAffinity (OXER1)	ΔAffinity (GPR35)	ΔAffinity (SUCR1)
CID 3822	3.053	0.597	0.05	-1.101	3.595
NuBBE 580	7.593	0.735	0.203	-2.32	3.055
CID 5479529	2.91	3.717	0.188	-1.169	2.83
NuBBE 582	2.743	0.915	-0.573	-1.318	2.85
NuBBE 1572	5.62	2.272	-1.79	-0.484	7.005
CID6758/ NuBBE 1414	4.863	3.351	-0.63	-5.107	2.457
NuBBE 1145	12.435	4.523	-0.493	-2.344	2.857
NuBBE 1106	6.302	-0.207	-0.384	0.068	4.356
CID31703	15.088	4.16	-0.459	-3.23	10.07

Table S7. Pocket volume, surface area, and depth of the orthosteric site in the active and inactive conformations of HCAR1 as calculated by DoGSiteScorer.

State	Volume (Å^3)	Surface (Å^2)	Depth (Å)
Inactive	2041.54	2624.81	41.67
Active	983.81	1179.53	23.54

Table S8. Solvent-accessible surface area (SASA) analysis for lactate and GHB and their analogs in the active and inactive conformations of HCAR1. SASA values were computed using Discovery Studio Visualizer v21.1.0.20298.

Ligand	Conformation	SASA (Receptor, Å²)	SASA (Ligand, Å²)	SASA (Complex, Å²)	ΔSASA (Å²)	Differential SASA (Å²)
Lactate	Inactive	15,047	94,261	14,940.90	94,367	-0.095
	Active	14,624	94,313	14,474.80	94,462	
2-acetylpropanoate	Inactive	15,047	116,999	14,949.60	117,096	-0.211
	Active	14,624	117,231	14,547.90	117,307	
prop-1-en-2-yl)oxy]propanoate	Inactive	15,047	137,243	15,029.40	137,243	0.809
	Active	14,624	136,325	14,497.30	136,452	
y-hydroxybutyrate	Inactive	15,047	109,832	14,926.30	109,953	2.520
	Active	14,624	107,397	14,588.70	107,433	
5-acetylpentanoate	Inactive	15,047	132,230	14,912	132,365	-1.626
	Active	14,624	133,875	14,508	133,991	
4-(prop-1-en-2-yloxy)butanoate	Inactive	15,047	149,663	14,974.60	149,735	
	Active	14,624	145,625	14,463.40	145,786	3.950

Figure S1. Predicted binding modes of Ketanserin (CID 3822) in the inactive (A–B) and active (C–D) conformations of the HCA1 receptor. (A) The 2D interaction diagram generated using PoseView (ProteinPlus platform) shows hydrogen bonds with Arg240$^{6.55}$, Tyr268$^{7.43}$, and Glu166 (ECL2), along with hydrophobic contacts with Leu150$^{4.60}$ and Ala96$^{3.33}$ (B) The 3D representation highlights the ligand, which is deeply buried in the orthosteric site and stabilized by Arg240 (2.2 Å) and Tyr268. Arg99 is shown for reference only to illustrate the absence of close polar contacts across conformations. (C) In the active conformation, the ligand forms weaker, more external interactions with Tyr149$^{4.59}$, Glu153$^{4.63}$, and Glu171$^{5.33}$, located above the orthosteric site. (D) 3D pose illustrates reduced binding depth and polar interaction density, consistent with lower affinity for the active conformation and predicted antagonist activity.

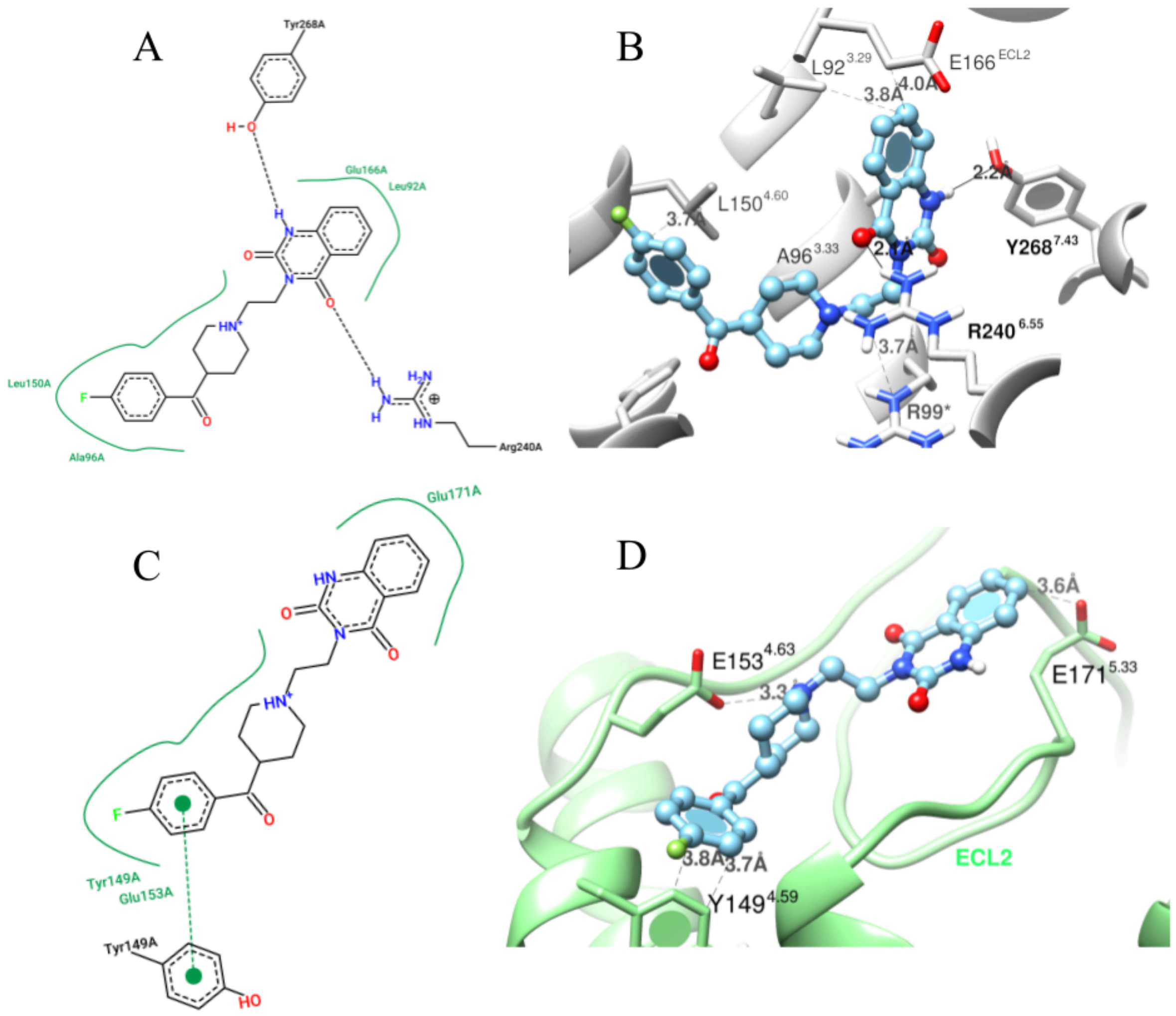

Figure S2. Binding interactions of Cryptopyranmoscatone A1 diacetate (NuBBE 580) in the inactive (A–B) and active (C–D) conformations of HCA1. (A) PoseView-generated 2D interaction map reveals hydrogen bonding with Arg240$^{6.55}$, Tyr268$^{7.43}$, and hydrophobic packing with Leu150$^{4.60}$, supporting deep orthosteric engagement. (B) 3D structure confirms the anchoring of the ligand via strong interactions with Arg240 (2.2 Å). Arg99 is shown for comparative reference only, with no direct polar interactions observed. (C) In the active state, the ligand transitions toward a less buried pose, interacting with Asn174$^{5.36}$, Glu153$^{4.63}$, and Tyr149$^{4.59}$(D) 3D rendering illustrates fewer deep interactions and less stabilization, consistent with antagonist-like behavior.

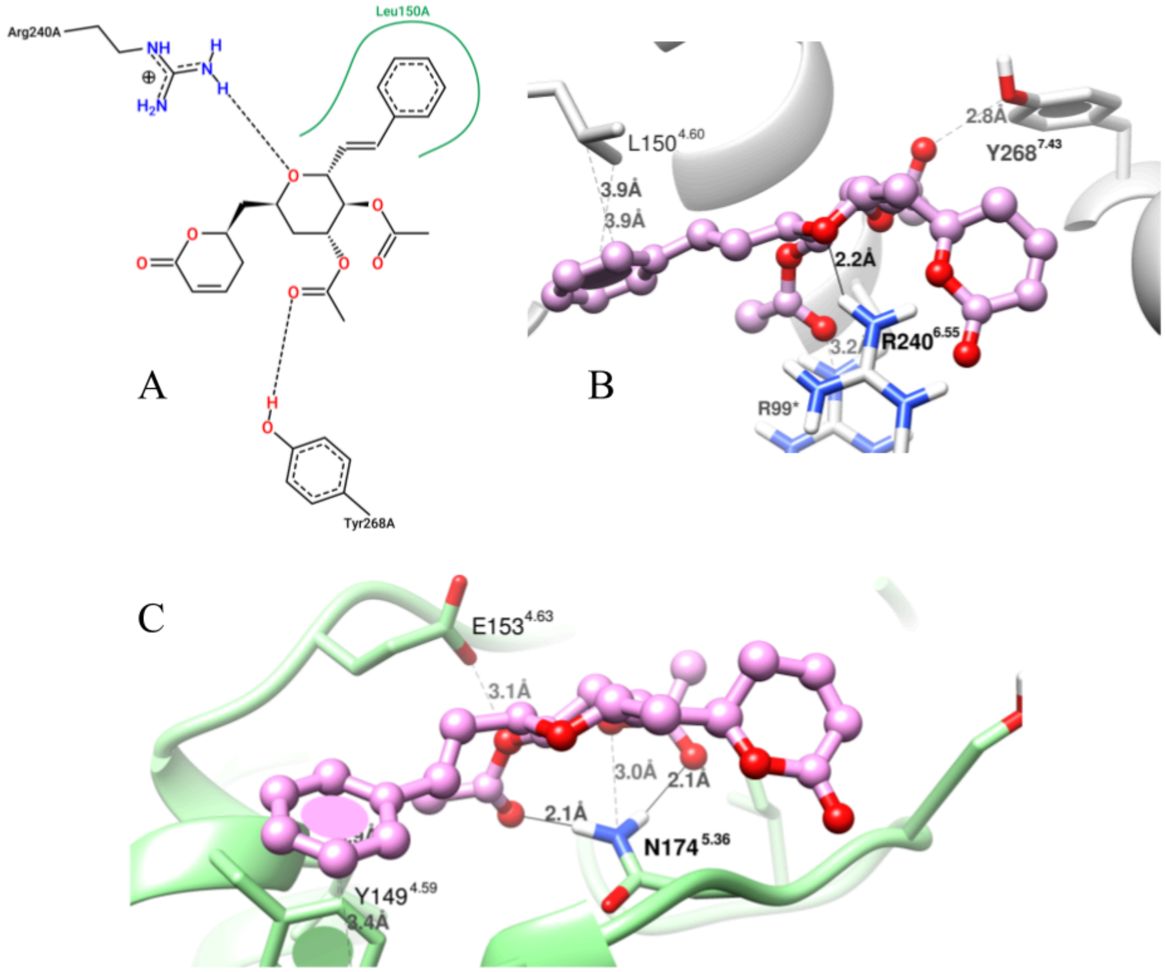

Figure S3. Predicted binding modes of Cefuroxime (CID 5479529) in the inactive (A–B) and active (C–D) conformations of HCA1. (A) 2D interaction map (ProteinPlus PoseView) shows a rich hydrogen bond network involving Arg240$^{6.55}$, Glu184$^{5.46}$, Gly146$^{4.56}$, and Tyr268$^{7.43}$, indicating strong binding within the inactive-state orthosteric pocket. (B) 3D view highlights close interactions with Arg240 (2.0 Å) and Glu184. Arg99 is included solely for reference, with no salt bridge or polar contact observed in either conformation. (C) In the active conformation, the ligand interacts with Asn174$^{5.36}$ and shifts outward toward ECL2. (D) 3D pose reveals fewer and shallower polar contacts, reinforcing reduced affinity for the active state and preferential stabilization of the inactive conformation.

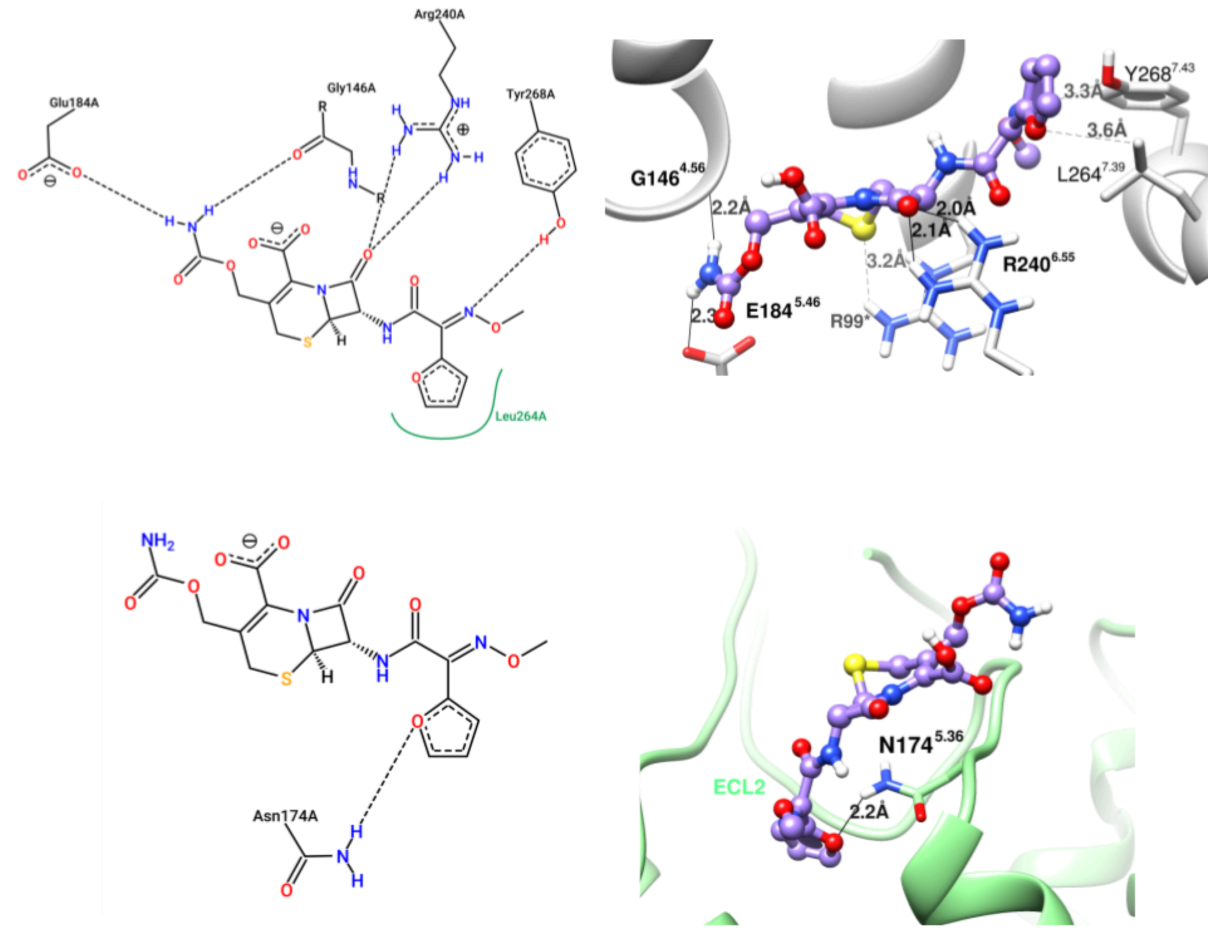

Supplement: Supplementary file 1 [file ao5c09253_si_001.pdf]
